# Dynamic soil columns simulate Arctic redox biogeochemistry and carbon release during changes in water saturation

**DOI:** 10.1038/s41598-024-83556-4

**Published:** 2025-01-24

**Authors:** Erin C. Berns-Herrboldt, Teri A. O’Meara, Elizabeth M. Herndon, Benjamin N. Sulman, Baohua Gu, Dawn M. Klingeman, Kenneth A. Lowe, David E. Graham

**Affiliations:** 1 Biosciences Division, Oak Ridge National Laboratory, Oak Ridge, TN 37831 USA; 2 Climate Change Science Institute, Oak Ridge National Laboratory, Oak Ridge, TN 37831 USA; 3 Environmental Sciences Division, Oak Ridge National Laboratory, Oak Ridge, TN 37831 USA; 4 Present Address: University of Wisconsin – Green Bay, Green Bay, WI 54311 USA

**Keywords:** Carbon cycle, Cryospheric science, Climate and Earth system modelling, Biogeochemistry, Climate-change ecology, Microbial ecology, Hydrology

## Abstract

Thawing Arctic permafrost can induce hydrologic change and alter redox conditions, shifting the balance of soil organic matter (SOM) decomposition. There remains uncertainty about how soil saturation and redox transitions impact dissolved and gas phase carbon fluxes, and efforts to link hydrobiogeochemical processes to ecosystem-scale models are limited. This study evaluates SOM decomposition of Arctic tundra soils using column experiments, water chemistry measurements, microbial community analysis, and a PFLOTRAN reactive transport model. Soil columns from a thermokarst channel (TC) and an upland tundra (UC) were exposed to cycles of saturation and drainage, which controlled carbon emissions. During saturation, an outflow of dissolved organic carbon from the UC soil correlated with elevated reduced iron and decreased pH; during drainage, UC carbon dioxide fluxes were 70% higher than TC fluxes. Intermittent methane release was observed for TC, consistent with higher methanogen abundance. Slower drainage in the TC soil correlated with more subtle biogeochemical changes. PFLOTRAN simulations captured experimental trends in soil carbon fluxes, oxygen concentrations, and water contents. The model was then used to evaluate additional soil water drainage rates. This study emphasizes the importance of considering hydrologic change when evaluating and simulating SOM decomposition in dynamic Arctic tundra environments.

## Introduction

Soil organic matter (SOM) in Arctic tundra soil decomposes to produce greenhouse gases through processes controlled by changes in soil moisture and aeration, which are difficult to replicate and simulate^1–3^. Permafrost environments represent a major reservoir of SOM, and gas fluxes are a major source of uncertainty in ecosystem-scale models simulating carbon dynamics^4^and global-scale coupled climate-carbon projections^5^. While water content and dissolved oxygen (DO) concentrations in Arctic soils vary seasonally, climate warming and permafrost thaw will drive regional hydrologic changes bringing dramatic transitions to these soils^6,7^. Water drainage resulting from permafrost thaw and melting ground ice is expected to promote drying of Arctic tundra^8,9^, but ground ice melting also causes land subsidence and thermokarst formation, saturating soil in drainage channels and driving the formation of thermokarst lakes^10,11^. Experimental warming studies^12^and seasonal geospatial monitoring^13^indicate land subsidence across the Arctic, with thermokarst representing about 20% of land coverage in the northern permafrost region^14^. Shifts in soil moisture associated with thermokarst formation control DO, redox biogeochemistry, and the rates and magnitudes of SOM decomposition to carbon dioxide (CO_2_) and methane (CH_4_)^15,16^.

Arctic ecosystem-scale models that simulate terrestrial carbon decomposition do not currently incorporate the effects of dynamic changes in soil redox state^17,18^. Current Earth system models simulate northern high-latitude soil carbon stocks with high uncertainty, suggesting opportunities to better model belowground processes that are affected by hydrology^19^. Improving estimates of terrestrial Arctic carbon cycling will require simulating redox-associated SOM mobilization and microbial decomposition mechanisms associated with changing soil moisture.

As soils and their microbial communities transition between saturated and drained conditions, different processes drive SOM mobilization and decomposition. Well-drained soils supply oxygen to microorganisms for aerobic respiration of SOM to CO_2_. Drained conditions also allow more efficient gas diffusion to the atmosphere, limiting the accumulation of CO_2_ and CH_4_in the soil column^20,21^. Increased water saturation drives greater soil pore connectivity and substrate transport, but also slows oxygen diffusion into the soil column and greenhouse gas diffusion to the atmosphere^22^. Under oxygen-limitation, anaerobic microbial processes such as fermentation, iron reduction, and methanogenesis become energetically favorable^3^, exerting strong controls on pH and redox potential, which may further destabilize protected soil carbon^23,24^. Organic carbon can be released from mineral surfaces at low or high pH due to electrostatic repulsion, and dissolved organic carbon (DOC) can be mobilized – or released to the aqueous phase – during reductive dissolution of iron mineral species^25–27^. Changes in redox potential and oxygen availability also cause changes in the microbial community composition of Arctic tundra soil and permafrost^20,28^. While reactive transport models for soil have been significantly enhanced to include biological processes, parameterizing mechanistic models to accurately simulate SOM degradation and greenhouse gas emissions under changing hydrology remains a great challenge^29–31^.

Efforts are underway to couple process-rich biogeochemical reactive-transport models to ecosystem-scale models to reduce uncertainty in carbon cycle simulations^31^. But there are still limitations in representing depth-dependent soil redox processes, including gaps in representing chemical species transport between soil layers, temporal variation in redox gradients, dynamic boundary conditions with permafrost thaw^32^, and biogeochemical hot spots^20^. Experimental studies can provide mechanistic insight and consistent data sets for model parameterization and validation. Previous field and laboratory studies have explored the influence of water content and oxygen availability on Arctic soil respiration at the plot scale^33–35^and batch microcosm scale^36,37^, but few Arctic soil decomposition studies have evaluated the column scale^38^. Soil column studies are needed to assess vertical transport, identify depth-resolved biogeochemical hotspots, and induce soil drainage.

The aim of this study was to quantify redox biogeochemical controls on Arctic tundra SOM decomposition in a vertical soil column during saturation and drainage. Soil cores from two different tundra topographic positions in Council, Alaska –upland core (UC) and thermokarst channel core (TC)– were compared to evaluate the impacts of pre-existing environmental conditions. Drainage cycles were expected to cause higher CO_2_ fluxes, but saturation cycles could mobilize additional SOM through reductive processes, with more mineral-associated SOM mobilized in upland soils that have not experienced continuous saturation. Specific hypotheses include: (1) CO_2_ gas fluxes decrease under saturated conditions, but dissolved CO_2_ accumulates in the soil column, decreasing pH, (2) increased SOM mobilization is associated with dissolution of iron mineral species, and (3) microbial communities from historically oxidized soils promote greater CO_2_ fluxes from aerobic respiration and less iron reduction and methanogenesis than those adapted to anoxic conditions. These hypotheses are interconnected: dissolved CO_2_ can contribute to mineral dissolution, which may release mineral-protected SOM and make it more bioavailable for microbial decomposition.

We used soil column experiments and reactive transport simulations to evaluate these hypotheses. Soils from both topographic positions were characterized for geochemistry and microbial community composition. Columns were packed with homogenized soil and cycled through drainage, saturation, and repeated draining phases. Temporal changes in column headspace gas concentrations (CO_2_ and CH_4_) were monitored in addition to soil porewater pH, ferrous/total iron, DOC concentrations, and aromaticity from specific UV absorbance (SUVA) measurements at five different depths. Soil columns were also instrumented with optical oxygen (O_2_) sensors and volumetric water content (VWC) sensors to link draining and saturation cycles to soil aeration and moisture. After the experiment, soils were again characterized for geochemistry, iron mineral phases, and microbial community composition. Microbial community data was used to prioritize biogeochemical transformation processes for modeling and interpret chemical measurements. Data collected from column experiments were used to parameterize a reactive transport model developed in PFLOTRAN^39^that included both aerobic and anaerobic decomposition reactions catalyzed by prominent microbial taxa in the soils. This reactive-transport model couples unsaturated flow equations with a biogeochemical reaction network^31,40^. Simulations evaluated how variation in soil hydrological properties, namely saturated hydraulic conductivity, influence SOM decomposition and transport. These preliminary simulations represent an important step toward coupling process-based, vertically-resolved redox biogeochemical models to ecosystem scale models. Decreasing uncertainty in simulations of terrestrial carbon cycling associated with oxic and anoxic transitions could support improvements to ecosystem scale models that estimate greenhouse gas fluxes and simulate future climate scenarios.

## Results and discussion

### Biogeochemical depth gradients confirm more reduced thermokarst soil

**Fig. 1:**
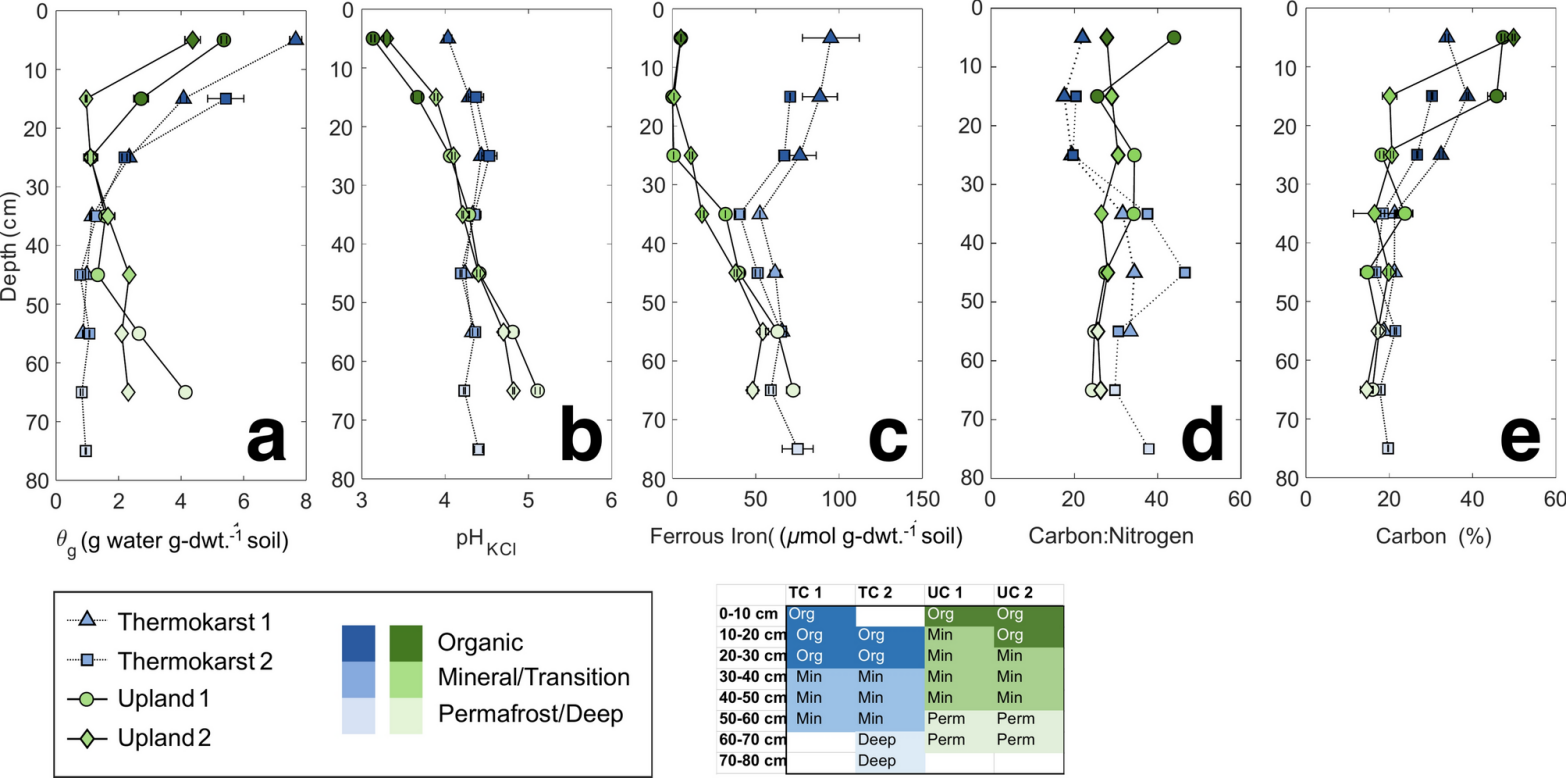
Soil core depth profiles for in situ thermokarst soil (blue squares and triangles) and upland soil (green circles and diamonds) show gravimetric water content **(a)**, pH extracted with 2 M KCl **(b)**, ferrous iron concentration from 2 M KCl extractions **(c)**, total carbon: nitrogen ratios **(d)**, and soil carbon composition **(e)**. Based on the geochemical depth characterization, soil depths were assigned to be organic soils (darkest marker colors), mineral/transition soils, or permafrost/deep soils (lightest marker colors). Depth profiles were characterized prior to processing for experimental setup. Thermokarst 2 was shifted down 10 cm to account for assumed soil loss in the field during sampling. Triplicate soil samples at each depth for each core were evaluated. Shading in the table indicates depth increments of cores that were later homogenized for soil column experiments.

Near-surface thermokarst soil was more water-saturated, more reduced, and less acidic than adjacent upland soil. Figure 1 presents depth profiles for soil properties, with the second thermokarst soil profile shifted down 10 cm to account for missing organic soil. Water content (Fig. 1a) is a major control on other soil properties, and the gravimetric water content (θ_g_) in these soils ranged widely from 0.95 to 5.37 g-water/g-dry weight (dwt) in the upland soil and from 0.77 to 7.67 g-water/g-dwt in the thermokarst soil, usually decreasing with depth. Wet Arctic tundra soils have relatively high θ_g_values due to permafrost-inhibited drainage and high organic matter content^41^. The higher θ_g_ near the surface (0 to 30 cm) in the thermokarst soil (blue markers) was associated with the submerged thermokarst channel, and the higher θ_g_ at greater depths (40 to 70 cm) in the upland soil (green markers) was associated with permafrost ice.

There was a greater range of pH and ferrous iron concentrations from surface to depth for the upland soil (Fig. 1b), potentially due to less vertical mixing of porewater relative to the saturated thermokarst soils. pH measured by KCl extraction (pH_KCl_) was lower at shallow depths in the upland soil, which may be due to accumulation of dissolved CO_2_, organic acids, or iron oxidation^3^. Higher pH at greater depths near the permafrost could be controlled by fewer root exudates, iron reduction, and accumulation of base ions. Increasing pH with depth is common in Arctic tundra soils^42–44^. Ferrous iron concentration measured in the top 30 cm was approximately 6 to 10 times greater in the thermokarst soil than the upland soil (Fig. 1c), indicating more reduced conditions in the saturated, thermokarst channels. Anaerobic respiratory processes, including biological iron reduction, consume protons and contribute to higher soil pH. Previous work has shown that iron cycling can be a major control on metabolic processes in Arctic soils^45^. Iron reduction could explain observations of higher pH near surface for the thermokarst soil, but this trend in pH depth profiles could also be due to more vertical mixing of pH buffering organic compounds in the thermokarst channel porewater.

Soil depth profiles of carbon and nitrogen illustrate compositional differences in these organic-rich upland and thermokarst soils. Upland soil profiles show a sharp drop in carbon (C) from 48% in the surface organic-rich horizons to 15% in deeper horizons (Fig. 1e). The thermokarst C profiles decrease gradually from 32% near the surface to 18% at depth. The C:N ratio of the upland soil varies from 24 to 44 (Fig. 1d), with higher ratios closer to the surface, consistent with herbaceous peatland soils^46^. Interestingly, the thermokarst soil has a higher C:N below 30 cm depth, potentially indicating buried peat with higher C:N or transport of relatively younger SOM to greater depths in the thermokarst channel. The %C and C:N ratios are comparable to other northern peatland soils^46^, and the soils in this study indicate peat composition at depths exceeding 30 cm. Soil depth profiles informed division of the soil cores into three sections for column experiments: 1) organic, 2) mineral/transition, and 3) permafrost/deep. Sectioning of the soils for the column experiments was informed by depth profiles presented in Fig. 1, and specific trends used to support soil sectioning include the following: high ferrous iron and organic carbon in the top 30 cm of TC, low %C and water content in deeper portion (60–80 cm) of TC, very high organic carbon in top 20 cm of the UC, and elevated pH and higher ferrous iron in permafrost soil (50–70 cm) of UC.

Upland and thermokarst soils included substantially different community structures of Bacteria and Archaea. The Faith phylogenetic diversity was higher in thermokarst samples than upland samples (Kruskal–Wallis, p-value = 0.015). Upland soils microbiomes contained high proportions of Proteobacteria, Actinobacteriota, Acidobacteriota and Bacteriodota phyla, while thermokarst soils were diversified with more Chloroflexota, Planctomycetota, and Verrucomicrobiota phyla as well as archaeal taxa (Figure S1). Bickel and Or proposed a model for soil microbial diversity based on habitat fragmentation in soils with disconnected pores^47^. The lower hydraulic conductivity observed in our thermokarst core would predict greater phylogenetic diversity according to this model. Deep soil samples (40–50 cm) generally had lower phylogenetic diversity than upper layers and lower species evenness, indicating substantial differences in the relative abundance of species. Permafrost from deep upland soils was dominated by only two phyla, Protebacteria and Actinobacteriota.

Microbial community populations were significantly different between the upland and thermokarst soils. PERMANOVA analysis of unweighted UniFrac distance metrics identified a significant difference in β-diversity between the topographic locations (UC and TC), independent of depth (p-value = 0.001) (Figure S2). The best predictors of bacterial/archaeal β-diversity distance in upland soils were pH and soil layer (Figure S3). This result is consistent with a previous report from a nearby Council site of pH and depth control on microbial community composition in surface soils of upland tundra^48^. In contrast, the best predictors of bacterial/archaeal β-diversity distance in thermokarst soils were carbon and water content, which correlated positively (Figure S4). In addition to the predominant Proteobacteria, Actinobacteriota, and Acidobacteriota phyla described above, there was a higher relative abundance of Bacteriodota and Domibacterota families in deeper upland soils, and Chloroflexota, Halobacteriota, Desulfobacterota, Caldisericota, and Thermoproteota taxa in thermokarst soils (Figure S1). Fungal diversity was much greater in upland soils than thermokarst soils, reflecting saprotrophs, endophytes, and ectomycorrhizal fungi summarized in the Supporting Information (Figures S5-S7). The different microbial communities in the upland and thermokarst soils reflect the different biogeochemical conditions in the wet upland tundra and the inundated thermokarst channels.

### Soil water drainage rate controls O_2_ availability, CO_2_ fluxes, and microbial activity

**Fig. 2:**
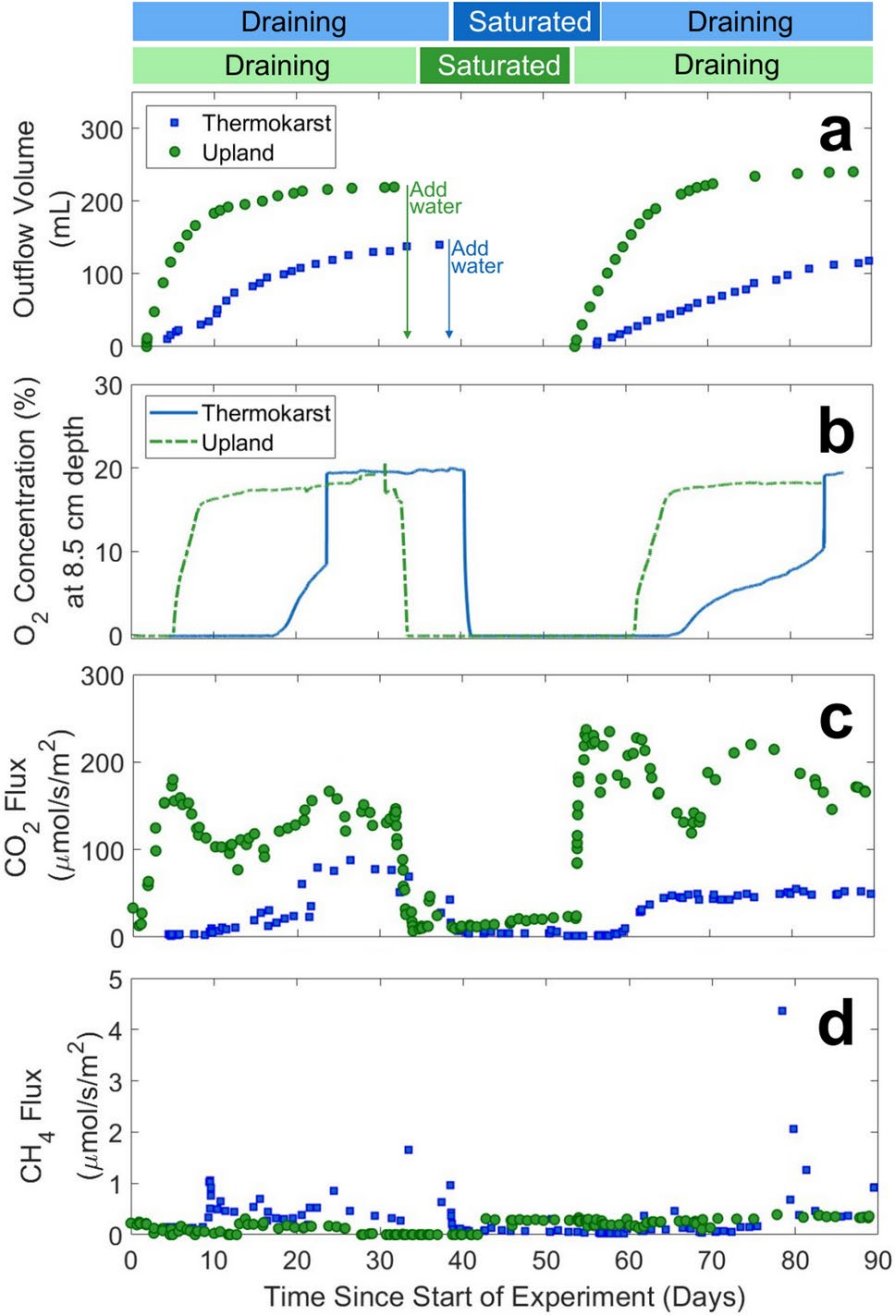
Experimental timeseries over the three-month column experiments with data from the upland column shown in green and the thermokarst in blue. Color bars across the top indicate periods of draining and saturation for each experiment. Outflow volume represents the cumulative volume of water leaving the base of the columns during draining phases, and decreases occur during water addition in saturation phases **(a)**. Oxygen concentrations (% volume) measured with an optical sensor at 8.5 cm depth are presented as an example of oxygen concentration variability **(b)**, with data from other depths presented in the Supporting Information Figure S9. Discrete headspace gas samples were used to determine fluxes of CO_2_ **(c)** and CH_4_ **(d)** during the experiments.

Two soil column experiments were conducted to evaluate effects of complete water saturation and draining on oxygen concentration (O_2_), and carbon dioxide (CO_2_) and methane (CH_4_) fluxes. Timeseries of outflow volume, O_2_ at a depth of 8.5 cm, and CO_2_ and CH_4_ fluxes for thermokarst and upland column experiments are presented in Fig. 2. Soils saturated at the beginning of experiments dried through an initial draining phase (~ 35 days), were flooded again and remained wet through a saturation phase (~ 20 days), and finally experienced a second draining phase (~ 35 days) (Fig. 2a). During the saturation phase, the outflow volume dropped to zero when the outflow port was closed, and deionized water was added to the top of the soil column. While the rapid addition of water at the top of the column was faster than would be expected for precipitation events, it may be more relevant for snowmelt and surface runoff events or abrupt subsidence events forming theromokarsts followed by saturation. While abrupt subsidence, such as thaw slump, is less likely to be observed at the site in Council, Alaska because permafrost thaw at Council is relatively gradual with slow geomorphological changes, other Arctic regions are experiencing more rapid land subsidence^10^. The rate of water outflow from the upland column was approximately four times the rate of the thermokarst column during initial draining (Figure S8), which was likely controlled by a lower hydraulic conductivity in the thermokarst soils. Lower hydraulic conductivity may be associated with an accumulation of finer soil particles in the thermokarst channels and relatively higher porosity in the upland soil^49^. These differences in outflow volume, likely associated with different hydraulic conductivities, had a major impact on the time each column was exposed to oxygen.

The faster drainage rate in the UC soil was also reflected by the volumetric water contents (VWC) of unsaturated soil horizons. In the upland column, VWC rapidly decreased at the surface (2 cm) during the first draining phase, followed by slower decreases at 15 and 27 cm (Figure S9), consistent with the outflow curve (Fig. 2a). Similar decreases in VWC were recorded during the second draining phase, except for a muted decrease at 27 cm that could be due to an increase in bulk density during operation. The VWC of unsaturated soils in the thermokarst column decreased gradually at 2 cm and 15 cm during the first draining phase, with a slow rate of change at 27 cm depth. In the second draining phase, the VWC decreased slowly at 2 and 27 cm, but dropped rapidly at 15 cm, consistent with the outflow curve. The rate of water outflow and decrease in VWC suggests that water drained quickly from high porosity organic-rich soil layers in the upland column, but more slowly from the low porosity layers of the thermokarst column and mineral-rich layers deep in both columns. VWC remained high (> 50%) at all layers during the experiments, probably associated with high carbon contents of 15 to 47% (Fig. 1). VWC measurements at different depths confirmed progression of the experimental dewatering front through the soil column and provided guidance for parameterizing simulations.

Rates of water drainage controlled oxygen concentrations in both soil columns (Fig. 2b). In the upland soil column, the O_2_ concentration at 1 cm depth increased quickly at the start of both drainage phases (Figure S9). O_2_ concentrations at 8.5 and 18.5 cm depth lagged the near-surface soil concentrations but increased quickly to near-saturation as VWC decreased and soil pores drained. Soils at all depths showed complete consumption of oxygen within a couple hours of re-saturation. In the thermokarst soil column, O_2_ concentrations increased abruptly during the first draining period, but were delayed in the second draining period. Slower water drainage was associated with soil column oxygenation at later times (Fig. 2b) and a delay in aerobic microbial respiration. O_2_ concentrations were negligible at the bottom of both columns for most of the experimental period, indicating a saturated, anoxic zone at the base.

CO_2_ fluxes in column headspaces showed lower fluxes under saturated conditions. The fluxes positively correlated with soil O_2_ concentrations and negatively correlated with water saturation (Figs. 2c and S10). Average CO_2_ fluxes during draining were greater in the upland column (140.8 µmol/s/m^2^) than the thermokarst column (33.9 µmol/s/m^2^), with maximum CO_2_ fluxes approximately 2 to 3.5 times greater for the upland column. CO_2_ fluxes in the upland column increased at earlier times during draining relative to the thermokarst column. These CO_2_ fluxes are much greater than fluxes observed in opaque field chamber experiments (0.37 to 5.44 µmol/s/m^2^) at the Council site^50^; this discrepancy may be due to the experimental design steps to homogenize soil, the lack of vegetation, exclusion of daily temperature fluctuations, or continuous purging of column headspace with air. Increasing CO_2_ fluxes preceded O_2_ increases at 8.5 cm for both soil columns, indicating that the shallower, organic-rich soil within the top several centimeters significantly contributed to CO_2_ fluxes, probably through aerobic respiration. For both draining phases, the upland column CO_2_ fluxes initially rose, decreased, and then increased again. Two potential explanations may describe these trends in CO_2_ fluxes for the upland column: 1) there was an initial release of accumulated CO_2_ near the air–water interface due to draining, followed by increasing aerobic respiration rates, or 2) there was an initial increase in aerobic respiration in the topsoil layer that quickly utilized labile DOC, followed by a release of CO_2_ derived from anaerobic decomposition deeper in the soil profile. In contrast, fluxes from the thermokarst soil column increased gradually before reaching a plateau. Both columns released low, but significant, amounts of CO_2_ during saturated periods, probably due to anaerobic respiration, fermentation, and acetotrophic methanogenesis. CO_2_ flux results confirmed expectations that lower CO_2_ releases would occur under saturated conditions.

CH_4_ fluxes demonstrated a more complex relationship with saturation and drainage than CO_2_ (Fig. 2d). Pulses of CH_4_ were observed in the thermokarst column around 35 and 80 days, when deeper parts of the column drained, VWC declined, and O_2_ penetrated the soil column. CH_4_ emissions were lower and less frequent during the second draining period. This could be driven by trapped CH_4_ being released when pores in mineral and deep soil layers drained. These pulses of CH_4_ indicate that ebullition, rather than diffusion, was the primary mode of CH_4_transport from deep soil layers to the column surface; prior work has also highlighted the important role of ebullition in permafrost soils^20,51^. CH_4_ emissions were low during the saturation period, although the column was fully anoxic and anaerobic methanogens were predicted to be active. For this relatively short-term experiment, significant negative correlations (p < 0.001, Figure S10) were observed for CO_2_ and soil saturation, but not for CH_4_ and soil saturation. Previous studies have shown that long-term incubations, up to several years, are sometimes required to observe significant accumulation in CH_4_from permafrost soils^52^. Only trace amounts of CH_4_ were released from the upland soil column during the experiment, though the discrete sampling approach used for gas measurements was unable to capture all pulses of CH_4_ released.

Relative rates of microbial respiration could have also contributed to timing and magnitudes of CO_2_ and CH_4_ fluxes. Microbial community “memory effects”, reflecting adaptation to prior environmental conditions, likely contribute to the higher CO_2_fluxes observed for the upland column under aerobic conditions. Similar microbial memory effects were observed in a field study that flooded portions of a drained thaw lake near Barrow, Alaska^33^and in laboratory incubations of soil cores from the Barrow Environmental Observatory^53^. Previous soil drying-rewetting experiments observed selection for both different species and traits within species to adapt to new hydrological conditions^54^. Prior adaptation of the thermokarst soil microbial community to anaerobic conditions, could also explain the observed pulses of CH_4_ in the thermokarst column. While the rate of water drainage controls oxygen availability in the soil column, the microbial community dynamics and history may simultaneously exert important controls on the total magnitude of CO_2_ and CH_4_ fluxes.

### Water and oxygen availability linked to changes in Fe, pH, and DOC

**Fig. 3:**
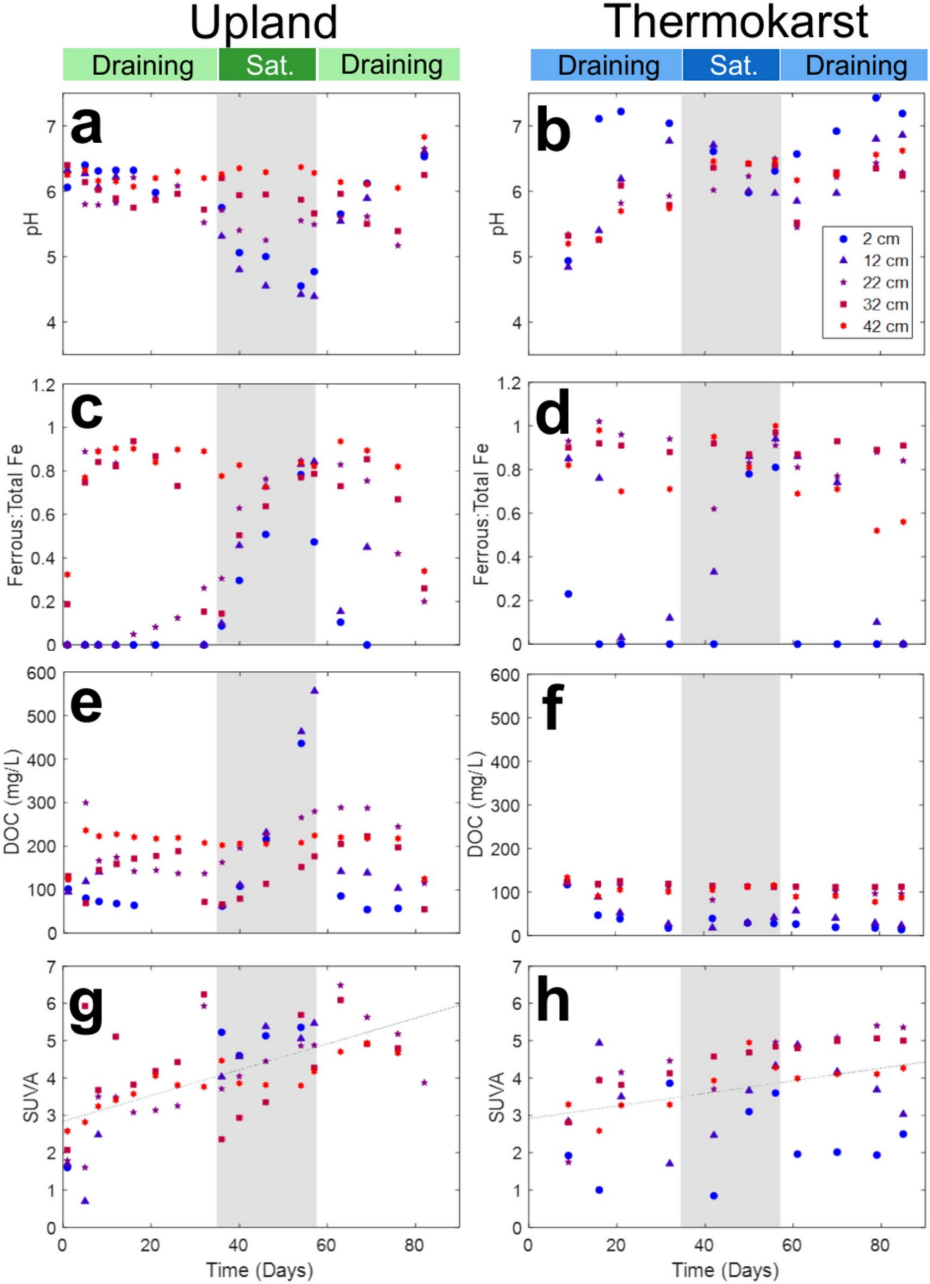
Experimental timeseries of discrete aqueous samples from Rhizons in column sample ports over the three-month column experiments. Different depths are indicated with different colors and symbols (cooler colors near the top of the soil column and warmer colors at the base). Data include pH **(a and b)**, the ferrous:total iron ratio **(c and d),** dissolved organic carbon (DOC) **(e and f), and** specific UV absorbance (SUVA) **(g and h)** for the upland and thermokarst columns. Grey areas indicate the period of soil water saturation.

Increased soil saturation coincided with lower pH and higher Fe(II)/Fe_total_ ratios at shallow depths in both upland and thermokarst columns (Fig. 3 and Figure S11). pH decreased at 2 cm and 12 cm depths (circle and triangle markers) for both columns under saturated conditions. In the upland soil column, pH dropped from 6.4 and 6.3 at 2 cm and 12 cm depths to pH values of 4.6 and 4.4, whereas the thermokarst soil pH at 2 cm and 12 cm decreased from 7.2 and 6.7 to 6.0 and 5.85, respectively during the saturation phase. Slight decreases were also observed at 22 cm for the upland column, likely due to more rapid soil draining. At deeper soil layers in both columns, pH increased slightly during the saturation phase. The surface soil pH decrease could be associated with CO_2_ accumulation during saturation, but accumulation of organic acids from microbial fermentation could also contribute. These results indicate especially large increases in soil acidity because organic-rich Arctic soils are known to have high pH buffering capacity^55^. The porewater Fe(II)/Fe_total_ ratios increased at most depths in the upland column during saturation but remained low in shallow soil depths in the thermokarst column (Fig. 3c and 3d). More than 75% of the dissolved iron in the thermokarst column remained reduced throughout the experiment, as the high proportion of reduced iron did not substantially change from its starting condition. Although biological iron reduction occurred in both columns during saturation, it was not the dominant pH control, because protons are consumed during this reaction. Under these conditions, most dissolved iron forms complexes with organic matter. While it appears that iron reduction did not control pH on the timescale of these experiments, it may have contributed to soil carbon release through alternative mechanisms. The lower pH and elevated ferrous iron under saturated conditions confirmed expectations that CO_2_ would accumulate in the soil profile, potentially contributing to the stability of iron minerals.

DOC and SUVA of porewater were measured to evaluate changes in porewater carbon concentration and aromaticity during soil saturation and drainage. DOC was chosen for evaluation, as it is assumed to be more bioavailable for mineralization than particulate or mineral associated carbon^56^. SUVA is the absorbance of a sample at 254 nm normalized by the DOC concentration, and it is a proxy for aromaticity of dissolved species water. DOC substantially increased during the saturation phase for the upland soil. At depths of 2 cm and 12 cm DOC increased by 374 and 400 mg/L, respectively, and DOC at depths of 22 cm and 32 cm increased by 103 and 86 mg/L, respectively (Fig. 3e). This dramatic increase in DOC under saturated, anoxic conditions was not observed for the thermokarst soil (Fig. 3f). The limited change in TC DOC concentrations could potentially be explained by the thermokarst channel historically being saturated and anoxic for extended periods of time and having already released labile SOM as DOC. Initial DOC concentrations in the UC soil ranged from 95–131 mg/L, representing the upper end of DOC concentrations reported in similar Arctic soils (5–150 mg/L)^57–60^, but concentrations as high as the maximum of 556 mg/L observed under saturated conditions in the column have not been reported in field studies. It is possible that reductive dissolution of iron minerals released bound organic carbon under saturated conditions in the upland soil^45,61,62^ contributing to the higher DOC concentrations; both columns had positive correlations with DOC and Fe(II)/Fe(III) in porewater (Figure S10). Increased SOM hydrolysis and fermentation may have also contributed to increases in DOC during saturation. The release of relatively labile DOC under anoxic, saturated conditions could have promoted aerobic respiration in the following draining cycle causing the higher CO_2_ flux observed for the upland column during the second draining phase (Fig. 2c). It is uncertain if additional oxic-anoxic cycling would continue to release pulses of DOC and fuel aerobic respiration in the upland soil, or if this phenomenon would only occur following initial saturation. SUVA for both upland and thermokarst column porewaters increased over time (Fig. 3g and 3h), indicating that the aromaticity of DOC increased in both columns during the experiment. This increase in SUVA was previously observed with other Arctic soil incubations^63^. This may be evidence for more labile, less aromatic, compounds being preferentially utilized by the microbial community^64,65^, or it could indicate that more aromatic compounds were released from SOM over time. The observed changes in DOC provide insight into the relationships between iron-carbon interactions and indicate that under saturated conditions more carbon is released to soil porewater, potentially contributing to elevated microbial respiration in subsequent draining cycles.

### Organic-bound iron transport in upland soils and oxidation in thermokarst soils

**Fig. 4:**
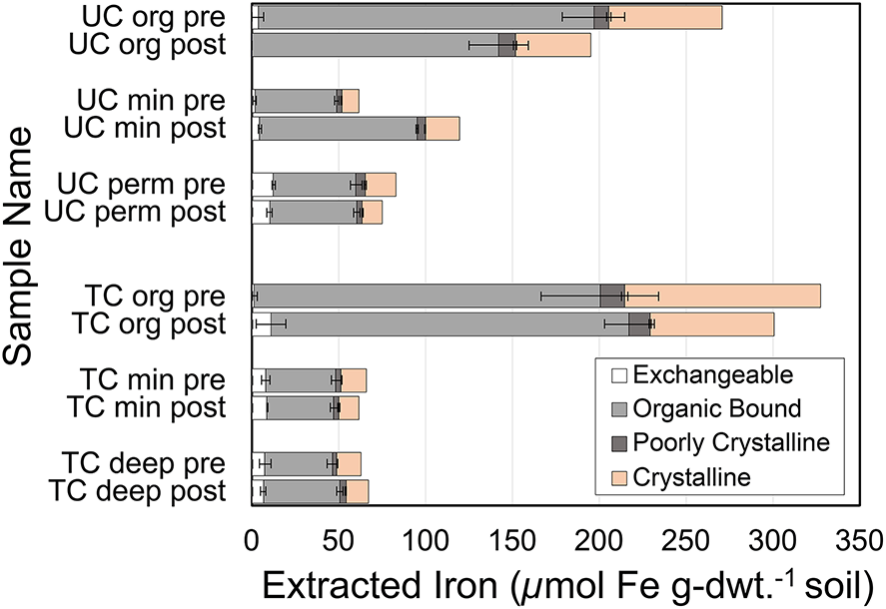
Extracted iron from the upland (UC) and thermokarst (TC) columns comparing soil Fe concentrations before the experiment (pre) and after the experiment (post) for the three different homogenized soil sections (org = organic, min = mineral/transition, and deep = deep/permafrost). Details for extracting exchangeable, organic bound, poorly-crystalline, and crystalline iron are included in the Methods and Materials. Exchangeable, organic bound, and poorly crystalline extractions were conducted in triplicate, and mean values are shown with standard deviations. Only one sample was measured for the crystalline extractions.

Soil extractions targeting different iron mineral phases showed limited redistribution of iron phases in the thermokarst column but provided evidence for transport of organic-bound iron in the upland column. Figure 4 presents the pre- and post-experiment iron distributions among phases for the upland (UC) and thermokarst (TC) columns. Although the iron distribution did not change in thermokarst soil layers, organic-bound iron in the upland soil was transported to the mineral layer. During the experiment, organic-bound iron in the organic layer of the UC decreased by 51 µmol Fe/g-dwt. and the mineral layer increased by 44 µmol Fe/g-dwt. The mineral soil layer of the UC was the only depth that showed an increase in water extractable organic carbon (WEOC) between pre and post experiment measurements (Figure S12). The downward transport of iron may be correlated with carbon mobilization in the upland soil column. X-ray absorption near edge structure (XANES) and EXAFS region data (Figures S13 & S14), provided additional evidence that the iron in organic soils was more oxidized than in mineral soils for both experiments (Tables S4 and S5), which was expected with organic soils experiencing more oxygen exposure near the surface. Slight increases in iron oxidation state post-incubation were also observed for both organic and mineral thermokarst experiment soils, which supports the assumption that significant soil drainage will induce solid-phase iron mineral oxidation.

### Microbial community composition was preserved during water table manipulations

The microbial community compositions changed in each soil column during these experiments, but they clearly reflected the initial composition from each topographic site rather than converging on a shared microbiome. While changes in phylum-level relative abundances were observed during the experiments (Figure S1), there was no significant difference in overall bacterial/archaeal phylogenetic diversity or evenness between pre- and post-experiment samples (p-values = 0.48 and 0.59, respectively). PERMANOVA analysis of unweighted UniFrac distance metrics identified a small difference in β-diversity between timepoints for both bacterial/archaeal and fungal communities, although those differences were not significant (p-values = 0.11 and 0.18, respectively). Nevertheless, individual taxa demonstrated significant changes in relative abundance during the experiments. In upland soils, ANCOM-BC analysis identified greater than 3 log-fold increases in the abundance of Acetivibrionales, Caloramatoracea, Paludibacteraceae, and Geobacteraceae and greater than 4 log-fold decreases in Burkholderiacaeae and Pseudomonadaceae families (Table S3). Similar compositional changes were observed in the thermokarst column: Geobacteraceae, Nanopelagicaceae, Ruminococcaceae and Acetivibrionales families all had > 2 log-fold increases. These compositional changes are consistent with decreased aerobic respiration and increased anaerobic fermentation and iron reduction. These soils demonstrated a strong memory effect of microbial community composition despite similar flooding and draining treatments that exposed them to both oxic and anoxic conditions, though greater shifts are likely with longer timelines. Although it is well established that disturbances cause changes in microbial community composition^66^, there is also good evidence for an ecological memory where past states of a soil influence community composition changes^67^. The absence of lateral microbial transport between UC and TC soils in our experiments limited opportunities for new species invasion during flood-drainage cycles.

**Fig. 5:**
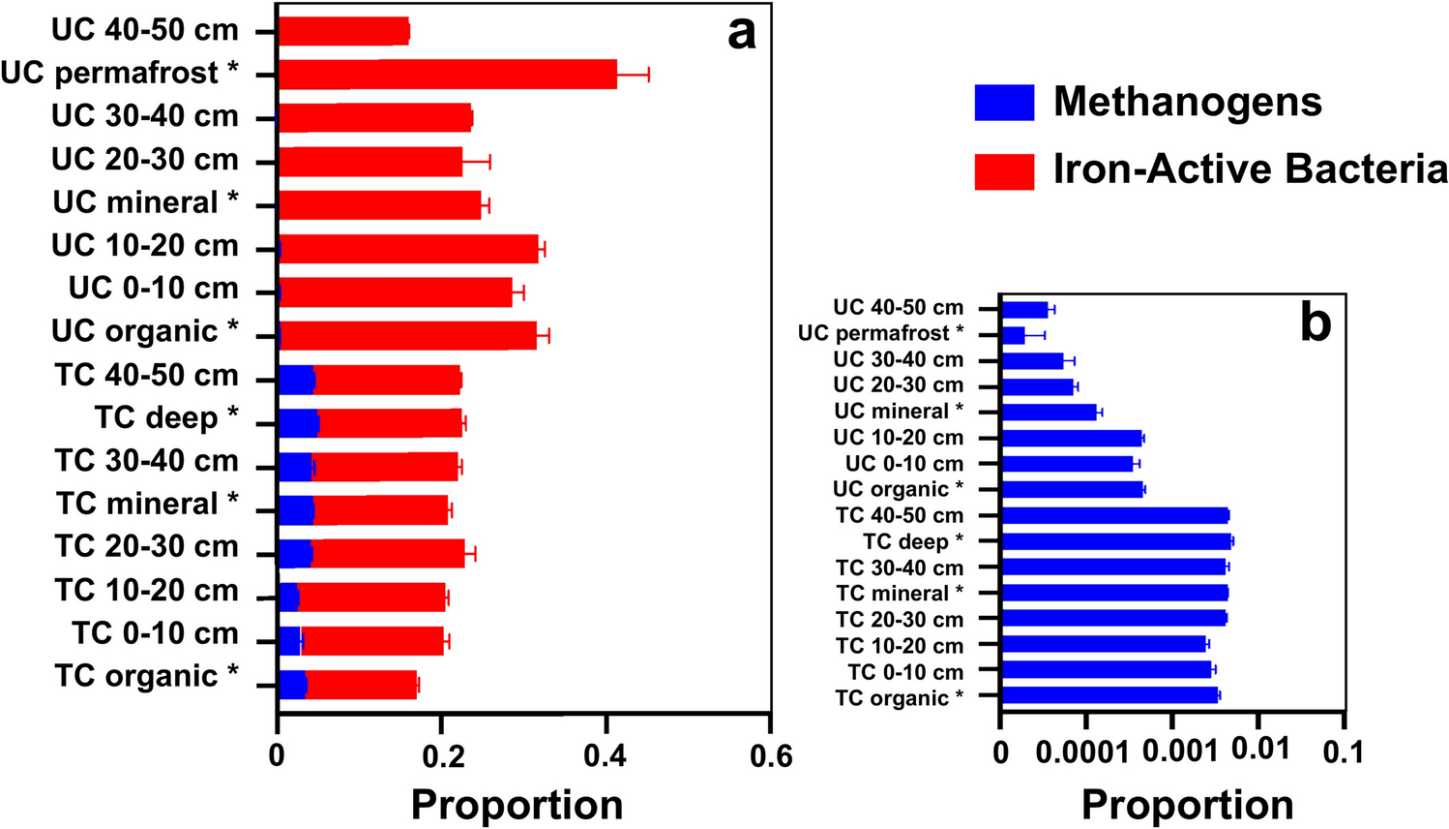
Relative microbial composition for methanogens and iron-active bacteria (part a), and log-scale plot for magnification of low relative abundances methanogens (part b). TC = thermokarst soil column; UC = upland tundra soil column. Org, Min, and Perm/Deep) = homogenized initial organic, mineral, and permafrost (deep) soil horizons, respectively. * Indicates initial soil microbial community pre-experiment. Depth increments (0–10, 10–20, 20–30, 30–40, and 40–50 cm) represent post-experiment community characterization along the soil column.

Additional analysis was conducted to evaluate the relative abundances of iron-active and methanogen genomes. The relative proportions of predicted iron-active bacteria were generally higher in the UC column than the TC column (Fig. 5), and the initial upland permafrost soil (UC-perm) had the highest proportion. Upland soils included iron oxidizing *Rhodoferax* and *Gallionella*, particularly in permafrost layers. Thermokarst soils contained both iron oxidizers and iron reducers, including Acidobacteriales, Koribacteraceae, Binataceae, Solibacter, and Acidobacteriota RHKY01 (Figure S15). Both abiotic iron oxidation and bacterial-mediated iron oxidation probably contributed to iron oxide formation during column draining, as observed in other Arctic tundra wetlands^68^. The relative abundance of methanogens was several orders of magnitude greater in thermokarst soils than upland soils, both before and after water table manipulations (Fig. 5). Methanogens were identified in all layers of the thermokarst soil column. Hydrogenotrophic *Methanobacterium* were most abundant in the top, organic soil, while acetoclastic *Methanosarcina* and *Methanothrix* were common in deeper layers. These methanogenic taxa have been frequently identified in wet Arctic soils, including previous studies near Council, Alaska^69,70^. Despite their low relative abundance, Methanobacteriales and Methanomassilicoccales were the primary methanogens in upland soils. Both acetoclastic and hydrogenotrophic methanogens were present in the soils, although the relative activities have not been determined. The consistent methanogen compositions in these soils suggest that low rates of CH_4_ production in the upland soil column during saturation were due to limited methanogen activity rather than an abundance of CH_4_ oxidizers. In thermokarst soils, CH_4_ cycling has the potential to be much more dynamic with methanogenic potential distributed throughout the soil column.

### Reactive transport simulations

**Fig. 6:**
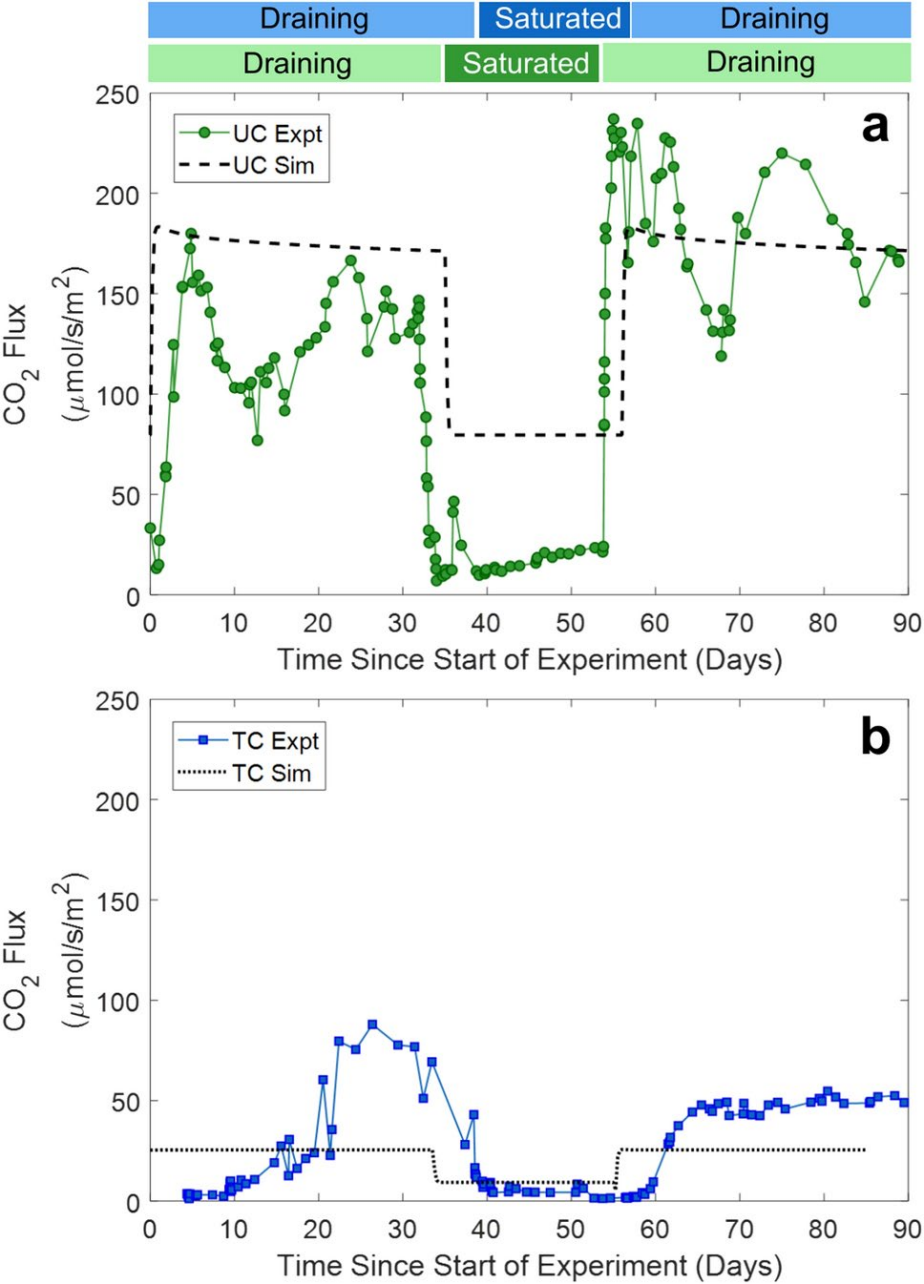
PFLOTRAN simulations compared to column experiment data for the upland column (UC) in part a and thermokarst column (TC) in part b. Headspace CO_2_ fluxes from the experiment (blue-colored markers) are plotted with PFLOTRAN simulation results (black dashed lines).

The coupled hydrology-biogeochemistry PFLOTRAN model was used to simulate multiphase reactive flow and transport in the upland and thermokarst soil columns, enabling multi-scale analysis and identifying opportunities for future improvements in parameterization and model structure. PFLOTRAN simulations effectively captured the trends in soil CO_2_ fluxes, despite underestimating the magnitude of TC fluxes overall and overestimating UC fluxes during saturation (Fig. 6). Simulations underestimated CH_4 _fluxes simulated relative to experimental measurements for the thermokarst column (Figure S16). This is likely due to relatively low assumed methanogen kinetics in the thermokarst column simulation or a lack of ebullition being represented in the simulation^71^. CH_4_ fluxes appeared linked to substrate concentrations in this model, and differences in initial conditions could have led to substrate depletion and limited methanogenesis in the simulations.

A sensitivity analysis for parameters controlling hydrology in the simulations was conducted to evaluate the extent to which saturated hydraulic conductivity (K_sat_), air and water diffusion coefficients (D_a_ and D_w_), and porosity (n) influence biogeochemical trends during saturation and drainage (Supporting Information, Extended Methods; Figures S17-S19). Overall, simulations confirmed that under saturated conditions, biogeochemical reactions are dominated by anaerobic processes (iron reduction and fermentation) leading to lower CO_2_ fluxes relative to drained conditions dominated by aerobic respiration. As expected, the draining phase of the experiments promoted higher CO_2_ fluxes. These model comparisons to the experimental column data are an important step towards integrating coupled hydrology-biogeochemistry in larger scale models.

**Fig. 7:**
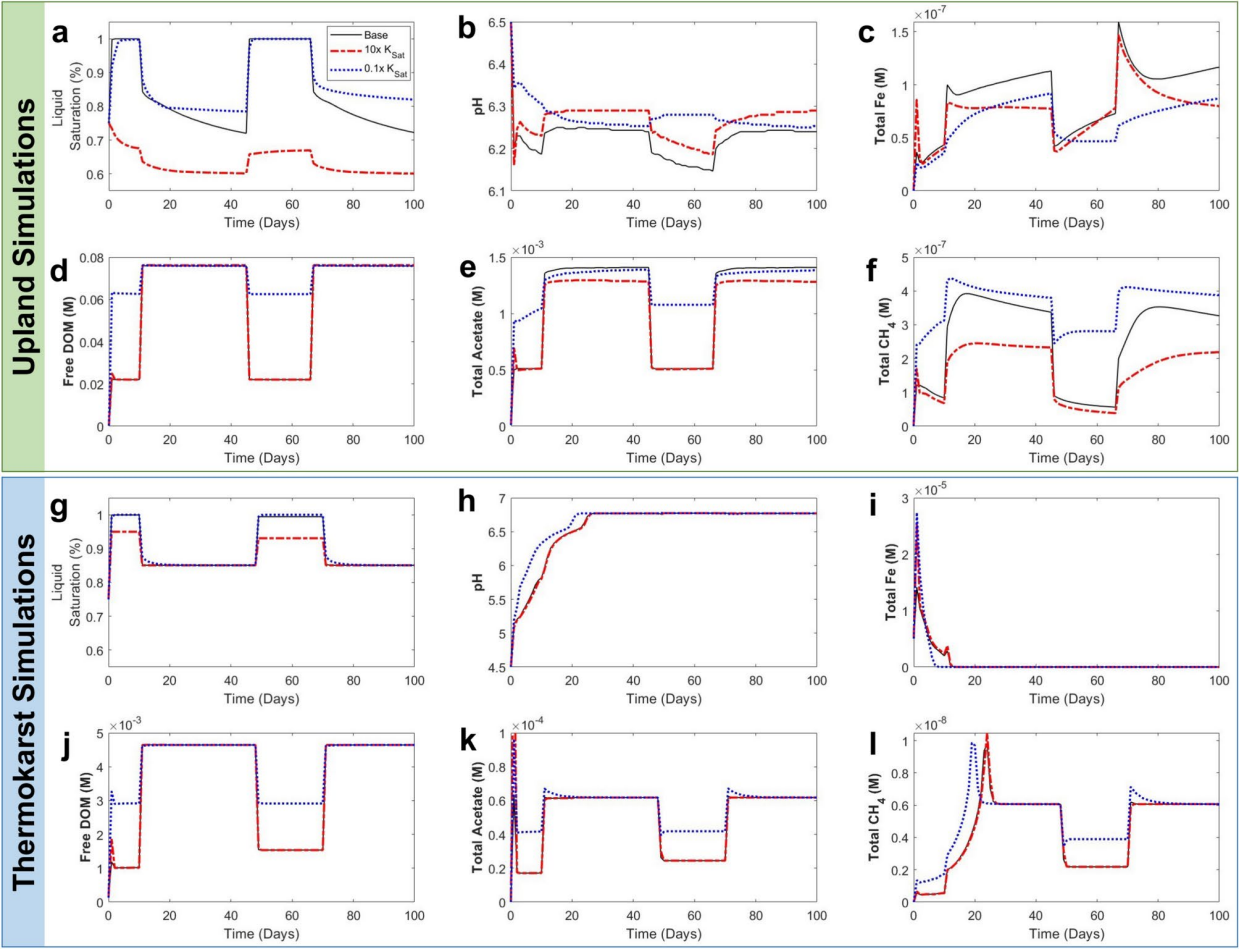
PFLOTRAN simulations for different saturated hydraulic conductivities (K_sat_) compared to the base case (black line). Increasing K_sat_ by an order of magnitude (10 × K_sat_, red dashed line) and decreasing by an order of magnitude (0.1 × K_sat_, blue dotted line) resulted in variations in response variables (liquid saturation, pH, Total aqueous Fe, Free DOM, Total aqueous acetate, and Total CH_4_) for both upland simulations (top, green outline) and thermokarst simulations (bottom, blue outline). Bold vertical axis titles indicate different vertical axis scales for upland and thermokarst simulations. Additional simulation results and a sensitivity analysis for the PFLOTRAN simulations are presented in Supporting Information.

Following model validation with the experimental Arctic soil column experiments, the sensitivity of the model to drainage rate was evaluated. Because K_sat_ is expected to be the most important control on the rate of water drainage from both upland and thermokarst columns, it was increased or decreased by an order of magnitude and resulting changes to response variables over time indicated the relative sensitivity. Figure 7 presents simulation outputs for response variables associated with different K_sat_ values relative to the base case (black line) over a 100-day timeframe. As expected, increasing the K_sat_ correlated with decreased liquid saturation for both upland and thermokarst simulations (Fig. 7a & 7g). Lower liquid saturation associated with faster draining – higher K_sat_ – was not directly correlated with increased oxygen concentrations (Figure S18), likely because UC microorganisms readily consumed oxygen, but did correlate with decreased free DOM (dissolved organic matter not associated with mineral phases), total acetate, and total CH_4_ (Fig. 7d-f & 7j-l). These simulations indicate that especially fast draining soils could be associated with less overall carbon mobilization.

Variation in pH between upland and thermokarst columns was quite different over the simulation timeline. The UC simulation (Fig. 7b), showed a transition from 0.1 × K_sat_ with relatively stable pH, slightly higher under saturated conditions, to higher variability in pH with 10 × K_sat_ where pH decreased approximately 0.1 pH units during saturation. Thermokarst column pH increased over the first 20 days and stayed relatively high for all three K_sat_ scenarios. These pH results are relatively consistent with the experimental results for pH (Figure S11), where the upland column which drained faster showed a greater decrease in pH than the thermokarst column, which drained more slowly. Simulation results may indicate an important relationship between high iron availability early in the simulation (Fig. 7i) and increasing pH (Fig. 7h), but further simulations focused on iron speciation, biotic/abiotic controls on iron mobility, and organic acid and dissolved CO_2_ availability would be necessary to holistically link iron chemistry to changes in pH. Based on the sensitivity analysis simulation results, K_sat_is a major control on pH, iron, and carbon cycling during draining of Arctic soils. Parameterization of diffusion coefficients (Figure S18) and porosity (Figure S19) also impacts biogeochemical response variables and should be considered more robustly in future analyses. Using soil hydrological properties in conjunction with known carbon loading and geochemistry will ultimately constrain estimates of soil carbon fluxes and improve models that simulate terrestrial controls on the carbon balance and future climate scenarios^8,72^. Emerging approaches for coupling reactive transport simulators into land surface models^40^ allow more sophisticated representation of carbon-geochemical interactions with the potential to improve projections of tundra greenhouse gas balance and its responses to a warming climate^5^, but are sensitive to hydrological properties. Our results highlight the value of improved soil hydrological properties for constraining uncertainty in biogeochemical model projections.

## Summary and conclusions

This study underpins the importance of considering transport and hydrologic change when studying soil carbon decomposition in dynamic Arctic tundra environments. Repeated cycles of saturation and draining modulated CO_2_ emissions from the upland soil column, with a smaller response from the thermokarst column. This hydrologic change caused an outflow of DOC and aromatic compounds from the upland column that was associated with pH changes, iron reduction, and mobilization from the organic to mineral layer. The decreasing pH is likely attributed to accumulating CO_2_ or organic acids in the soil porewater, which were then released during draining. Increased Fe(II)/Fe_total_ during saturation confirms the soils transitioned into reducing conditions, which could be fueling reductive processes that release Fe(III)- or Fe(III)-oxide-bound DOC in upland soils. That released DOC may act as a substrate for aerobic respiration during later draining. The slow-draining thermokarst soil column had relatively small changes, focused on the surface layer. The initial soil conditions, including microbial communities and SOM composition, affected the timing and magnitude of emissions and required separate parameterization for simulations.

While this study provides important data that will support development of reactive transport models that can be coupled to larger ecosystem scale models, it still has several limitations. The soil cores used in this study represent a single, low Arctic tundra site, and soils from other regions could have different hydrological and geochemical properties that would yield different results. Additional column experiments with a broader range of soils could allow better sampling of cross-site variability in responses to saturation and drainage. It is valuable to note that significant variability in maximum CO_2_ fluxes (234 µmol/m^2^/s for UC and 87 µmol/m^2^/s for TC) was observed in this study, despite not targeting carbon flux hot spots, such as drained thaw lakes or abrupt thaw slumps. This highlights the importance in understanding carbon dynamics in potentially lower carbon-emitting Arctic landscapes. Future work coupling hydrology and biogeochemistry in the Arctic also needs to adequately represent lateral transport. Drainage rates in this study may have been limited by a low permeability layer that, in the Arctic landscape, would have promoted lateral flow. This study also homogenized soil increments and neglected the important roles of Arctic vegetation. A limitation of homogenizing cores, such that enough mass was available for full instrumentation of the columns, was the disruption of the soil structure. Results from this study cannot quantitatively evaluate hydrologic drainage rates for each soil type, which is why experiments were designed as a comparison between two organic-rich Arctic soils. Despite these limitations, the current study makes an important step toward coupling biogeochemistry to changes in soil hydrology. The column experiments provide evidence for increased DOC concentrations when upland soils become saturated, potentially acting as an important source of labile carbon that is not yet represented in reactive transport models. The microbial analysis also highlights the important role of soil history in understanding the dominant microbial communities driving SOM decomposition, with historically saturated soils showing higher methanogen relative abundances, and carbon flux data supporting that historically aerobic soils more efficiently convert SOM to CO_2_. As greater need develops to accurately simulate the Earth system, terrestrial redox biogeochemistry will need to effectively be linked to regional hydrologic trends. PFLOTRAN simulations in this study provide one example of a modeling approach that could ultimately improve terrestrial components of Earth System Models. Future studies will need to continue developing efficient approaches to couple soil column scale biogeochemistry to regional hydrologic and atmospheric models to effectively account for terrestrial climate feedbacks and decrease uncertainty when simulating future climate scenarios.

## Materials and methods

### Field site description and sampling

**Fig. 8:**
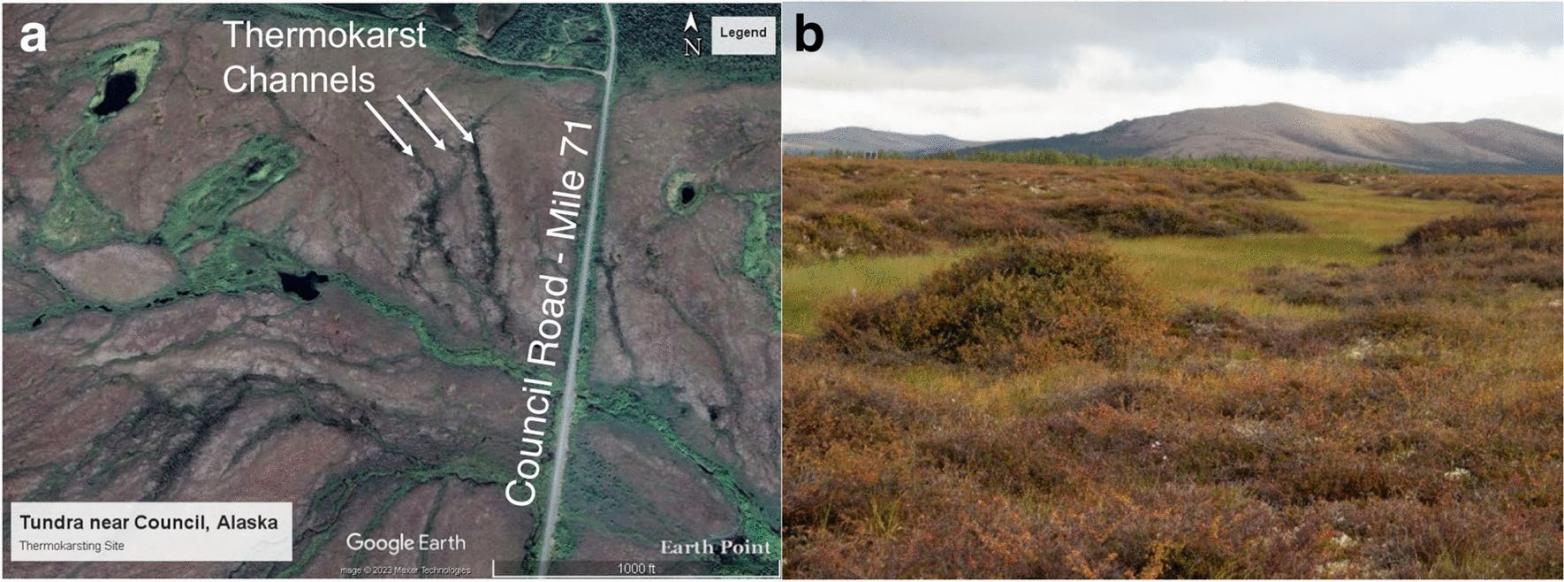
Research site near Council Road mile marker 71. Aerial view of site showing thermokarst channels **(a)**, and a ground-view of the thermokarst channels showing variation in vegetation from the upland with low shrubs and tussock to the thermokarst channels with relatively more sedges **(b)**. The channels are formed from thawing permafrost and land subsidence.

Gelisol soils were collected from the Next Generation Ecosystem Experiments (NGEE) -Arctic site near Council Road mile 71 in the Seward Peninsula of Alaska, United States. Thermokarst channels and lakes have developed at this site as a result of land subsidence promoted by thermal erosion and thawing permafrost. This area is representative of a large Bering Tundra ecoregion, which is predicted to spread to the Arctic tundra on the Alaskan North Slope by the end of this century^73^. Figure 8a shows an aerial view of the site, annotating some thermokarst channels that are the focus of this study. The thermokarst channels were inundated with water, whereas the adjacent wet, acidic upland tundra had a high volumetric water content (maximum near 0.6 m^3^/m^3^ in the summer, Figure S20), but soils were not inundated. Vegetation in the thermokarst channels was dominated by wet graminoid and moss plant functional types (PFTs), and the upland tundra was characterized by deciduous low shrub, dry graminoid, and lichen PFTs^74,75^. Figure 8b shows a thermokarst channel with the lighter green regions being the wet graminoids in the thermokarst channel observed in August 2021.

Tundra core samples were collected from the site in July 2018 and stored frozen. Upland cores were obtained using a modified SIPRE auger (Jon’s Machine Shop; Fairbanks, AK) with a Powerhead motor containing a sterilized polyvinyl chloride (PVC) liner. Replicate cores NGADG0189 and NGADG0183 were collected from the same location within a meter of each other (Lat. 64.859833 Long. −163.698213) in an upland area with a thaw depth of 63 cm. Both soil cores were 72 cm long with 3-inch diameters, and the core holes were 97 cm deep, reflecting compression of the litter and organic layers during coring. The surface soil volumetric water content was 49%. A 2-inch diameter gouge auger (AMS) was used to collect two cores, NGADG0184 and NGADG0185, from a water-saturated thermokarst channel (Lat. 64.860044 Long. −163.697718) with a thaw depth > 110 cm. The cores were 71 and 68 cm long, respectively, and were transferred to sterile liners in the field. All soil cores in liners were sealed, transported in blue ice-filled coolers, frozen, and shipped to Oak Ridge National Laboratory (ORNL) with dry ice. Soil cores were stored in a chest freezer at −20 °C prior to characterization and analysis.

### Soil characterization

Frozen soil cores were partially thawed for six hours in a vinyl, anoxic glove bag adapted to accommodate a core-length transfer chamber (Coy Laboratory Products). The chamber utilized a nitrogen atmosphere with 1–2% hydrogen gas. Soils were sectioned into 10 cm increments and thawed for another two hours (on ice packs to maintain low soil temperatures) before subsamples were taken from each increment to analyze water content, pH, iron concentrations, and both carbon and nitrogen in triplicate. Remaining soil from each increment was stored frozen until measurements were complete (approximately 1 week). Two cores each from the upland and thermokarst locations were processed and characterized in 10 cm increments. Three sub-samples from each increment were analyzed for each analysis.

Gravimetric soil water contents were analyzed by measuring mass before and after drying in a 105 °C oven overnight. Soil pH was determined via KCl extraction (pH_KCl_); this method produces more stable pH measurements but underestimates the pH relative to water measurements^76,77^. pH_KCl_ was determined in a slurry of 1 M KCl (1:5, mass:volume) with air-dried soil (n = 3) using an Accumet Basic AB15 bench pH meter with a double-junction pH/ATC combination electrode (Fisher Scientific). This system was calibrated between pH 4 and 10 at ambient temperature using buffer standards (Orion). Exchangeable iron was determined using a nitrogen sparged 1 M KCl extraction solution (1:10, m:v) that was shaken for 30 min under anaerobic conditions before analysis using the 1,10-phenanthroline colorimetric assay (HACH 8008 FerroVer for total iron and HACH 8146 Ferrous Iron assay for reduced iron). Absorbance was measured with a UV–Visible absorption spectrophotometer (Beckman DU800) at 510 nm. The total iron assay includes a reductant, and the Fe(II):Fe(total) ratio was determined from extracted Fe(II) and Fe(total) concentrations. Carbon and nitrogen were analyzed using a LECO TruMac CN macro determinator with soils that were dried at 105 °C overnight, stored in a desiccator, and then dried once more at 105 °C two hours before analysis. These measurements were used to develop depth profiles of the soil geochemical parameters and differentiate between similar soil depth increments.

### Soil column saturation-drainage experiment set up

A temperature-controlled column system was developed to monitor biogeochemical changes in soil porewater and associated greenhouse gas release during saturation and drainage events. Two separate experiments were conducted: thermokarst soil column (TC) and upland soil column (UC). The column consisted of a PVC tube (7.5 cm diameter, 60 cm long) with threaded ports (ORNL machine shop) for porewater sampling and sensor installation. A flange was attached to the base of the PVC tube, and a bottom plate with an outflow valve was bolted on the bottom. The bolts functioned as legs to support the column. A mesh screen was installed at the bottom of the column with 10 cm of sand overlying the screen to distribute outflow.

After geochemical characterization of the upland and thermokarst cores with depth, similar depth increments were homogenized to create three distinct zones (organic, mineral/transition, and permafrost/deep) for both the upland and thermokarst cores. The homogenization step was necessary to evaluate biogeochemical changes in the soil pre- and post-experiment; without homogenizing, it would be challenging to differentiate heterogeneity in the soil profile from true changes in biogeochemistry that occurred during saturation and drainage. Homogenization was conducted in an anaerobic chamber filled with nitrogen and 1% hydrogen (COY Laboratories), with soil increments mixed by hand using a sterile spoon in sterile bags on top of ice packs to minimize soil warming. The PVC tube for the column experiments was packed with homogenized soil increments inside the glove bag starting at the base of the column (above the sand layer) with the permafrost/deep soil and moving upward through mineral/transition, and organic soil zones. Soil was added in “lifts” of 100–200 g of wet soil, with slight compaction after each lift using a sterile stainless-steel rod with a flanged end. During the packing phase, column ports were covered with parafilm. The packed soil column was sealed, removed from the anerobic glove bag, and stored at 4 °C in an environmental chamber until experiment initiation the following day. This process was completed separately for the thermokarst and upland soil experiments.

**Fig. 9:**
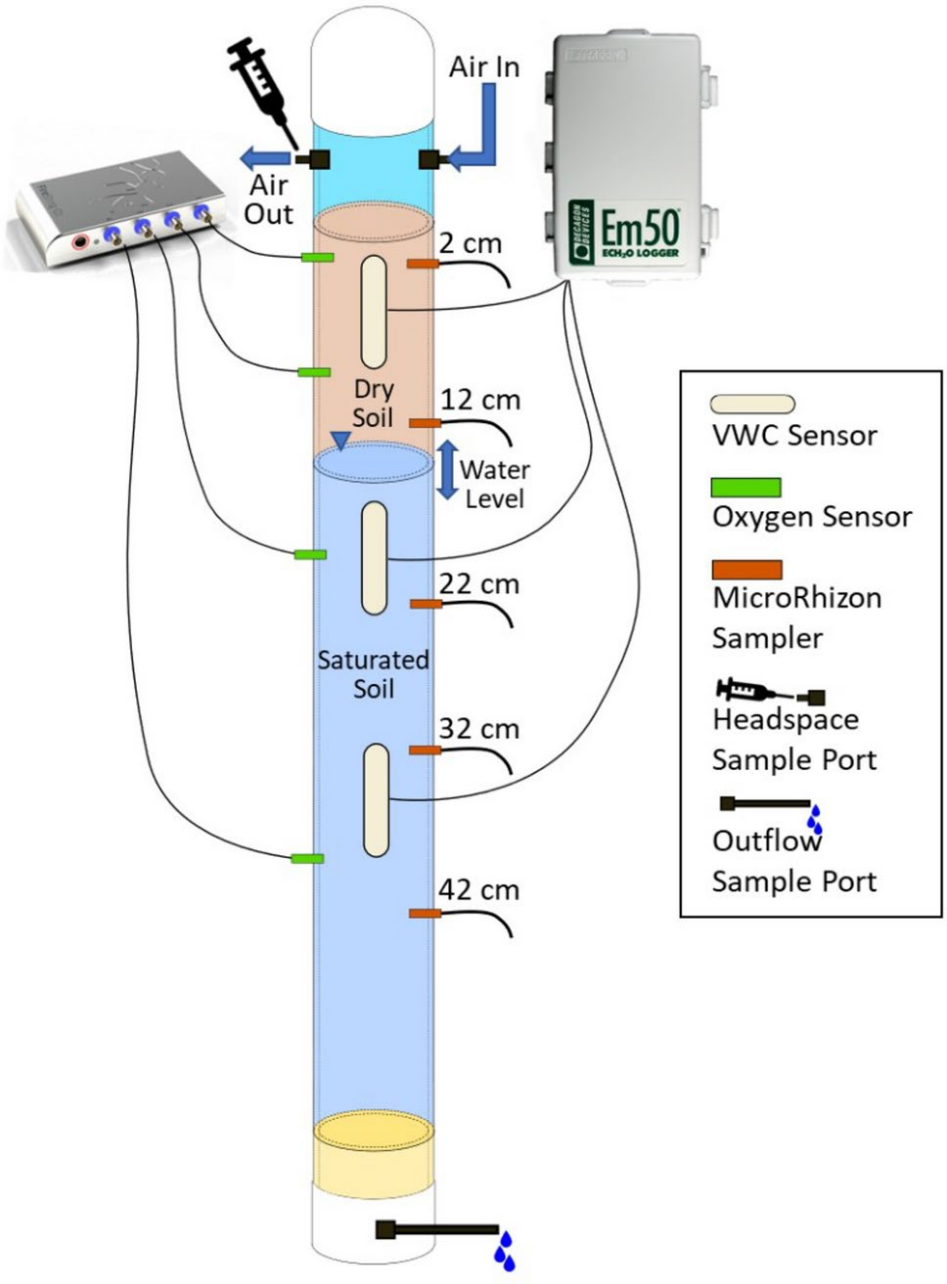
Soil column experiment with sampling ports for Rhizon samplers and sensors for water content and oxygen. Air was continuously pumped through the headspace at the top of the column. During draining phases, the bottom outflow sample port was open.

The column temperature control, sensor installation, and saturation were completed two days prior to the initial experimental draining phase. The soil column was maintained at 4 °C by wrapping the packed PVC column in copper tubing connected to a circulating cooling unit (PolyScience) filled with an antifreeze solution. Three volumetric water content sensors (Teros 12, METER) were installed at depths of 2 cm, 15 cm, and 27 cm from the top of the soil column and secured in place using large zip ties. Calibration curves for volumetric water content were pre-determined for organic and mineral soils using the same setup with the sensors inserted through a “calibration column” at known volumetric water contents. Parafilm covering sampling ports was replaced with compression fittings threaded into the ports before wrapping the column and copper flow lines with black nitrile butadiene/ polyvinyl chloride insulation (0.5 inch thick). Optical oxygen sensors (Pyroscience, OXROB3), were inserted at depths of 1 cm, 8.5 cm, 18.5 cm, and 36 cm (from the top of the soil) after pre-piercing the soil with a comparable diameter sterile stainless-steel rod, and then securing with compression fittings. A similar approach was used to install Rhizon porewater samplers (Eijkelkamp) at depths of 2 cm, 12 cm, 22 cm, 32 cm, and 42 cm. Figure 9 details the soil column sensor locations. After sensor installation and two days before the initial column drainage, distilled, deionized water (Millipore) at 4 °C was used to raise the water level in the soil column to the soil air interface. Thermocouples were used to confirm internal soil temperatures at depths of 36 and 8.5 cm during the 24 h following sensor installation and soil saturation.

### Column experiment operation and sampling

Column experiments were operated with a constant flow of 5 mL/min of air through the soil column headspace. The portion of headspace at the top of the capped soil column had an outlet port to allow continuous flow. Headspace gas mixing was modeled as a continuous stirred tank reactor (CSTR) with a constant inflow and outflow, and a source term was used to relate headspace gas concentrations to fluxes from the soil (Supporting Information, Equation S1).

The columns underwent three drainage-saturation phases during the experiment, and the TC and UC columns were evaluated for 94 days and 88 days, respectively. The initial draining phase began when the outflow port at the base of the column was opened (Fig. 9). The first phase lasted approximately 35 days (32 days for UC and 37 days for TC), controlled by the hydraulic conductivity of the thermokarst soil. Column effluent samples were collected and weighed to estimate the outflow rate over time. At the beginning of the second phase (the saturation phase), the outflow port was closed and distilled, deionized water was added over two hours to increase the water elevation in the soil column to the interface between the top of the soil column and the headspace gas. The top VWC sensor at 2 cm depth was used to confirm that the column stayed saturated. After approximately 20 days of saturation (21 days for UC and 19 days for TC), a second draining phase followed (28 days for US and 35 days for TC), again with effluent collection. Water levels in the soil column during the draining phases were controlled by gravity drainage, and the drainage rate was assumed to correlate with soil hydraulic conductivity.

Headspace gas samples were taken every one to four days, and small volumes of porewater at five different depths were sampled approximately once per week. Pre-evacuated, 12 mLvials (Exetainer, Labco, High Wycombe, UK) were used for headspace gas sampling; 12 mL of soil column headspace gas was sampled through the headspace outflow port and immediately injected into an Exetainer vial. Samples were stored upside-down in a layer of water to prevent any losses prior to analysis by gas chromatography (described below). The same Exetainer vials were used to sample soil porewater by inserting a needle from the Rhizon sampling line directly into the Exetainer vial, and allowing the vacuum in the vials to remove approximately 1.5 mL of porewater over 5 to 10 min. Vials were massed before porewater extraction and after to estimate total volume of sample by mass. A subsample of each porewater sample (200 µL) was removed and immediately evaluated for ferrous and total iron as described above. After measuring headspace gas in the porewater sampling vial and taking another aliquot for further water analysis (300 µL), pH was measured in the porewater samples. Effluent water from the soil column was collected from the outflow sample port during draining phases in 20 mL glass vials. Effluent samples were passed through an Acrodisc syringe filter (0.8 µm prefilter with 0.2 µm filter, Pall Corp.) and stored at 4 °C prior to analysis.

### Gas and aqueous sample analyses

Headspace gas samples from the soil column were analyzed using a gas chromatograph with a flame ionization detector (FID) and methanizer (Shimadzu GC-2014) to determine carbon dioxide (CO_2_) and methane (CH_4_). The GC instrument was also equipped with TCD and ECD detectors to monitor other gases, including higher CO_2_ concentrations and nitrous oxide (N_2_O). An AOC-6000 autosampler (Shimadzu) was used to inject samples from the Exetainer vials into the inlet port and gases were separated on a HaySepD column (5 m by 0.125 inch, 80/100 mesh). Greenhouse gas concentrations, CO_2_ and CH_4_, were determined from linear regression of a five-point calibration curve using five separate mixed-gas standards. Concentrations were converted to fluxes based on the cross-sectional area of the column, the volume of headspace gas, and the flow rate of air through the headspace volume (Supporting Information).

Two different types of aqueous samples were collected during the experiments. Soil porewater samples from each Rhizon sampling port were collected with pre-evacuated Exetainer vials, and column effluent samples were collected from the outflow port at the column base during draining phases (Fig. 9). Ferrous and total iron were analyzed in the porewater samples immediately after sampling using the 1,10-phenanthroline method described above. After measuring headspace gas (SRI GC-FID with methanizer) in the porewater sample vials and sub-sampling for anions and DOC, pH was measured with an InLab Micro pH combination electrode (Mettler Toledo). The remaining volume of the porewater sample was filtered (0.45 µm) and used to determine SUVA, measured using a Beckman DU 800 UV–Vis spectrophotometer in a quartz cuvette (Starna Cells). SUVA is the absorbance of a sample at 254 nm normalized by the DOC concentration.

Samples from both the Rhizon sampling ports and the column effluent were analyzed for dissolved organic carbon with a TOC-L analyzer with ASI-L autosampler (Shimadzu). Samples were filtered (0.45 µm) and then acidified (1% HCl) before analysis. Aqueous samples from the sampling ports were also analyzed for anions using ion chromatography with a Integrion ion chromatograph equipped with a AS15 analytical and guard columns (Dionex), a hydroxide eluent generator, and a conductivity detector. Targeted anions included major anions (chloride, nitrate, phosphate, and sulfate) and a suite of organic acids (formic, acetic, propionic, oxalic, pyruvic, and citric). Due to the small sample size (300 µL) and dilution required for analysis, limited information was available for the organic acids measured, as many were near the detection limit for this method.

### Soil composition and microbial community analysis

Soil carbon and nitrogen compositions were determined pre and post experiment for five different depths for both TC and UC experiments using a LECO TruMac CN or an Elementar Unicube. Samples were air-dried, ground with a mortar and pestle, and sieved (No. 200 sieve). Samples were then placed in an oven (105 °C) overnight, stored in a desiccator, and then dried once more at 105 °C two hours before analysis. LECO soil standards were used to quantify carbon and nitrogen percentages in soil samples.

Sequential soil extractions targeting different iron mineral phases were conducted pre and post experiment to evaluate impacts of saturation and drainage on redistribution of iron. The extraction protocol was based on previous studies^59,78^, and targeted water extractable, exchangeable, organic-bound, poorly crystalline, and crystalline phases. The following extraction solutions were used for each phase: water extractable iron with anoxic water, exchangeable iron with 0.1 M BaCl_2_-NH_4_Cl, organic-bound iron with 0.1 M Na_4_P_2_O_7_ (pH 10), poorly crystalline iron with 0.25 M NH_2_OH•HCl, and crystalline iron with bicarbonate-buffered sodium dithionite and citrate (0.11 M NaHCO_3_, 0.1 M Na_2_S_2_O_4_, 0.27 M Na_3_C_6_H_5_O). All extractions used 1 g of soil with 10 mL of extraction solution, and additional details are included in the Supporting Information.

The microbial community was evaluated pre- and post-experiment to explore shifts in the relative abundances during soil saturation and drainage cycles over the 90-day experiment. Samples were collected from initial homogenized depth increments (three homogenized soil types in triplicate for both experiments – TC and UC), and from post experiment soil (five different depths in triplicate for both TC and UC experiments). Soil samples were stored at −80°C until DNA extraction. The extraction protocol utilized a DNeasy PowerSoil Pro Kit (Qiagen) according to the manufacturer’s instructions with lysing by bead beating on a mixer mill (Retsch). DNA sequencing used the Illumina 16S metagenomic sequencing library preparation guide (Part 15,044,223 Rev B) to prepare amplicon metagenomic sequencing libraries. Pooled primers targeting the V4 region of the 16S ribosomal RNA (rRNA) gene for Archaea and Bacteria and the internal transcribed spacer 2 (ITS2) region for Fungi were used for amplification^79–81^. A Bioanalyzer with a DNA7500 chip (Agilent Technologies), was used to validate pooled libraries. The final library pool concentration was determined using a Qubit fluorometer with the broad range double stranded DNA assay (Thermo Fisher Scientific). A MiSeq instrument (Illumina) with V2 chemistry was used to complete a paired end sequencing run (2 by 251 by 8 by 8). Amplicon sequence data were deposited in the National Center for Biotechnology Information Short Read Archive with BioProject accession number PRJNA902031.

Data analysis for the DNA sequencing focused on understanding shifts in specific microbial communities. The QIIME 2 bioinformatics platform (ver. 2023.2.0) was used to analyze the demultiplexed amplicon sequence data^82^. Adapter sequences were removed from the paired-end sequences using cutadapt software^83^, and the sequences were denoised using DADA2^84^. Taxonomic classification of the 16S rRNA gene amplicon sequence variants (ASVs) used the Greengenes2 database^85^to train a Naïve Bayes classifier^86^, while fungal ITS2 AVSs were classified using the UNITE QIIME release for Fungi 2^87^. A holistic analysis was completed using QIIME 2 workflows: this included an evaluation of diversity indices (Kruskal–Wallis test), principal components comparison related to sample locations and biogeochemical measurements, and relative compositions of the microbial community. Permutational multivariate analysis of variance (PERMANOVA) tests were performed in the QIIME 2 environment using the adonis package of *vegan*^88^. Assumptions were then made linking specific genus and species with different metabolic processes (Supporting Information).

### PFLOTRAN simulations

A reactive-transport model was developed using PFLOTRAN^39^ and a biogeochemical reaction scheme modified from Sulman et al.^31^ to simulate current experimental conditions and differentiate hydrological controls from biogeochemical controls on CO_2_ and CH_4_ soil gas fluxes. Model simulations were used to evaluate if CO_2_ fluxes were more strongly related to changes in hydraulic conductivity or initial soil carbon content. While the model framework chosen requires parameterizing hydrology and chemical reactions separately, the simulations can still account for interconnections and feedbacks between these processes in natural environments. Details for the model development and sensitivity analysis are included in the Supporting Information, and model code is available on Github at https://github.com/omearata/REDOX-PFLOTRAN. In brief, a 1D soil column was simulated with outflow at the base to promote soil drainage. Experimental outflow data was used as a boundary condition to simulate water level changes in the soil column (Figure S21). The availability of water and oxygen in the soil column drives transitions in oxic/anoxic biogeochemical reactions in different layers of the model. Biogeochemical reactions included decomposition of dissolved organic matter, aerobic respiration, iron reduction and oxidation, and methanogenesis^31^. Initial conditions for the model simulations are described in the Supporting Information. In short, both the TC and UC columns were parameterized with organic soil properties from 0–15 cm and mineral soil properties from 15–50 cm (Table S1). Model parameters were adjusted to represent experimental conditions, and then a sensitivity analysis considered the following three scenarios (Table S2):

1. Scenario 1 – Changes in Drainage Rates: The saturated hydraulic conductivity of both organic and mineral soil layers was increased or decreased by an order of magnitude relative to a base case represented by experimental conditions. This simulation was used to evaluate how the rate of drainage can impact biogeochemical responses in Arctic soils.
2. Scenario 2 – Changes in Porosity: Both layers of soil were evaluated at a maximum (0.9) and minimum (0.1) porosity to decouple impacts of porosity on hydraulic conductivity.
3. Scenario 3 – Changes in Water and Air Diffusion Coefficients: The water/aqueous diffusion coefficient and the air/gas diffusion coefficient were either doubled (2x) or halved (0.5x) to evaluate the impact of diffusion on carbon transport and decomposition.

The scenarios evaluated were intended to evaluate how hydrological changes would impact biogeochemistry. Often higher carbon content is associated with higher K_sat_ and higher soil water retention, and the goal of the first scenario simulation was to decouple these controls. Soil carbon percent was kept constant while K_sat_ was increased or decreased. In most soils, changes in porosity are also linked to K_sat_ and drainage rates, and these simulations allowed for evaluation of higher and lower porosity without associated changes in K_sat_. Finally, because some low K_sat_ conditions may be more impacted by diffusive controls, the water and air diffusion coefficients were altered to evaluate associated biogeochemical changes and carbon transport. End members of the sensitivity analysis for K_sat_ were in a range possible for Arctic soils, whereas the porosity lower end (n = 0.1) was quite low and doubling or halving the diffusion coefficients from known diffusion coefficients at in situ temperatures is likely less realistic, but still an important consideration for overall transport processes in unsaturated soils.

## Supplementary Information

Supplementary Information.

## Data Availability

Data from the experiments described here is available online: Erin Berns, David Graham. 2022. Arctic Soil Column Saturation & Drainage Experiment for Biogeochemical Redox Dynamics; Council, Alaska 2020–2022. Next Generation Ecosystem Experiments Arctic Data Collection, Oak Ridge National Laboratory, U.S. Department of Energy, Oak Ridge, Tennessee, USA. Dataset accessed on 5 November 2023 at 10.5440/1875838. Amplicon sequence data were deposited in the National Center for Biotechnology Information Short Read Archive with BioProject accession number PRJNA902031.
