# Supplementary material for: Dynamic soil columns simulate Arctic redox biogeochemistry and carbon release during changes in water saturation
Source: Sci Rep. 2025 Jan 24;15:3093. doi: 10.1038/s41598-024-83556-4 (PMC11759714; doi:10.1038/s41598-024-83556-4)
Supplement: Supplementary file 1 — Supplementary Information. [file 41598_2024_83556_MOESM1_ESM.docx]

**Supporting Information:**

**Dynamic soil columns simulate Arctic redox biogeochemistry and carbon release during changes in water saturation**

E. C. Berns-Herrboldt^1,2†^, T. A. O’Meara^3,4^, E. M. Herndon^4^, B. N. Sulman^3,4^, B. Gu^4^, D. M. Klingeman^2^, K. Lowe^4^, and D.E. Graham^2,3^

^1^ University of Wisconsin – Green Bay; Green Bay, WI 54311 USA

^2^ Biosciences Division, Oak Ridge National Laboratory; Oak Ridge, TN 37831 USA

^3^ Climate Change Science Institute, Oak Ridge National Laboratory; Oak Ridge, TN 37831 USA

^4^ Environmental Sciences Division, Oak Ridge National Laboratory; Oak Ridge, TN 37831 USA

Corresponding author: David E. Graham ([grahamde@ornl.gov)](mailto:grahamde@ornl.gov))

† Former affiliation

# S1. Extended Methods

## S1.1 Volumetric Water Content Sensors Calibration

Teros 12 (METER) volumetric water content (VWC) sensors were used to monitor changes in soil column moisture at three different depths in the soil column experiments. Sensors were calibrated using a representative organic soil for the top sensor, and the lower two sensors were calibrated with a mineral soil. A small calibration column made of the same PVC material used for the column experiments was constructed, and ports were installed the same as they were in the experimental columns. The first calibration was conducted with *in situ* soil VWC, and the initial water content was determined before adding additional water for the higher saturation standards or drying for the lower saturation standards. A regression of sensor response to VWC was developed for both soil types and used to process temporal data from the Teros 12 sensors.

## S1.2 Calculation of Gas Fluxes from Soil

Headspace gas concentrations were measured for CO_2_ and CH_4_ and used to determine the flux (${mass}/{(area*time}$) of soil gas to the column headspace. The column headspace volume (V) had a constant inflow of air (Q) at 5 mL/min which resulted in a gas residence time in the headspace of approximately 2 hours. The headspace was assumed to act as a continuously stirred tank reactor (CSTR) according to Equation S1.

$$\frac{dC_{out}}{dt}=Q*\left( C_{in}-C_{out} \right)+F*A Equation S1$$

The outflow concentration (C_out_) is assumed to be the same as the fully mixed concentration in the headspace, and the inflow concentration (C_in_) is assumed to be atmospheric CH_4_ (0 ppm) or CO_2_ (420 ppm) concentrations. The flux term (F) was used as a source term fitting parameter to account C_out_ measured in the headspace of the column. The cross-sectional area (A) of the column was used to represent the area of the top of the soil column at the soil-air interface.

## S1.3 Soil Iron Extractions

The soil iron extraction protocol was informed by previous studies^1,2^, and targeted water extractable, exchangeable, organic-bound, poorly crystalline, and crystalline iron. Each extraction used 10 mL of extraction solution and 1 g of soil and were extracted in an anaerobic chamber (COY Laboratories). Details for each sequential extraction step are included below. Except for the crystalline extractions that were only conducted for one replicate, all other extractions were conducted in triplicate. After each extraction step, soil slurries were centrifuged at 4000 rpm (~3000 g) for 30 minutes and filtered through 0.45 µm syringe filter. The filtrate was reacted with a FerroVer iron assay (HACH 8008), and the absorbance was measured on a Beckman DU800 at 510 nm. Blanks were included for both the extraction solution and the extraction solution plus the FerroVer reagent to account for background absorbance due to the extraction solutions. The extraction solution for the water-extractable iron included anoxic water degassed with nitrogen and shaken for 90 minutes before analysis. Excess extraction solution from this step was used for water extractable organic carbon (WEOC) analysis. The second, sequential extraction solution for exchangeable iron was a barium ammonium chloride solution (0.1 M BaCl_2_-NH_4_Cl) degassed with nitrogen and shaken for 20 minutes before analysis ^3^. In the third extraction, organic-bound iron was extracted with sodium pyrophosphate (0.1 M Na_4_P_2_O_7_ pH 10) with shaking for 16 hours^4,5^. In the fourth extraction, poorly crystalline iron was extracted with hydroxylamine hydrochloride (0.25 M NH_2_OH•HCl pH 2) with shaking for 48 hours ^6^. In the final extraction, which was only completed for one sample at each depth, crystalline iron oxides were extracted with a bicarbonate-citrate-dithionite solution (0.11 M NaHCO_3_ + 0.1 M Na_2_S_2_O_4_ + 0.27 M Na_3_C_6_H_5_O) with shaking for 16 hours^2,7^. Data for extractions was reported as mass of iron extracted per gram of soil.

## S1.4 Extended X-ray Absorption Fine Structure (EXAFS) and X-Ray Absorption Near Edge structure (XANES) Analysis

Six samples were analyzed for iron K-edge x-ray absorption spectroscopy at beamline 9-BM at the Advanced Photon Source in March 2022. Pre and post experiment samples were evaluated for the thermokarst column and only pre-experiment samples were evaluated for the upland column. Dried soils were packed into sample holders and analyzed in fluorescence mode using an unfocused beam. Sample spectra were energy calibrated to an Fe foil (E0 = 7110.8 eV) and compared to a suite of reference compounds. Linear combination fits (LCFs) to the XANES region were conducted using reference spectra from single valence compounds (FeO, Biotite, siderite, FeS, iron(II)oxalate, iron(III)oxalate, ferrihydrite, lepidocrocite, goethite, hematite, and iron(III)citrate) to evaluate iron oxidation state. Combinatorial LCFs to the EXAFS region were used to determine iron speciation; standards used in combinatorial fits were down selected to include most relevant species for organic or mineral soils (i.e., iron(III)oxalate, ferrihydrite, biotite, chlorite, pyrite, and iron(III)citrate). A reference compound was not included in the fit if it did not improve fitting parameters by at least 10%. Iron compounds are reported in classes as ferrihydrite, organic-Fe(III), silicate Fe, and other Fe(II) rather than as specific compounds.

Figure S7 indicates that iron in organic soils was more oxidized than iron in mineral soils for both experiments, and both organic and mineral soils for the thermokarst soil column experiment showed slight increases in average iron oxidation state post-incubation (Table S4). Iron in organic soils was most similar in appearance to organic-bound iron and ferrihydrite, and iron in mineral soils was most similar to Fe(II) contained in silicate minerals or Fe(III) oxides (Figure S8, Table S5). Non-silicate Fe(II) components may be similar to pyrite (Table S5), but more analysis would be required to gain a robust identification. Iron speciation in the organic soil of the upland core was consistent with a high proportion of organic-bound Fe(III) (51%) whereas the organic soil in the thermokarst core contained relatively higher proportions of ferrihydrite (67%).

## S1.5 Microbial Community Analysis and Data Processing

DNA extractions were conducted pre and post experiment for both columns. Three different depths (to represent the three different homogenized layers) were evaluated pre-experiment, and five different depths were evaluated post experiment.

Fungal phylogenetic diversity was greater in upland than thermokarst samples (p-value = 0.04) (Figure S14). PERMANOVA analysis of unweighted UniFrac distance metrics identified a significant difference in β-diversity of fungal communities between the core samples (p-value = 0.001). The Ascomycota phylum was dominant in thermokarst soils (Figure S15), with a West Siberian bog fungus, *Ascocoryne turficola*, comprising the principal species^8^. Upland soils also contained abundant *Basidiomycota* (Figure S14). Prominent ecological functions predicted by mapping taxa to the FungalTraits database included saprotrophs, root endophytes, lichen parasites, and ectomycorrhizae in the upland soils, and dominant saprotrophs in the thermokarst soil^9^.

Metabolic functions predicted in the metagenomes were consistent with observed biogeochemical conditions. PICRUSt2 was used to predict physiological activities^10^, with results analyzed by ANCOM-BC for upland versus thermokarst soils. The upland soil microbiome had greater potential for hemicellulose degradation, lignin aromatics degradation, oxidative glucose degradation, tryptophan degradation, C_1_ oxidation, and TCA pathway activities. The thermokarst soil microbiome had greater potential for basic amino acid degradation (arginine, lysine, ornithine and glycine), propanediol degradation, methanogenesis, hydrogenase, fermentation, and purine degradation. The FeGenie tool predicted iron-active genomes in microbiomes based on relatively abundant taxa similar to microorganisms with known genome sequences^11^. Syntrophic bacteria from the Syntrophales family were also associated with iron(II)-rich soils.

## S1.6 PFLOTRAN Model Development, Parameter Fitting, and Sensitivity Analysis

A process-based model developed in PFLOTRAN was used to simulate experimental reactive transport using reaction chemistry after Sulman et al. (2022)^12^. The model was designed to use outflow volume data to approximate drainage in the soil column, and soil properties (saturated hydraulic conductivity, porosity, and carbon percent) controlled the soil water retention and rate of oxygen diffusion into different soil layers during draining. The simulations included organic (0-15 cm) and mineral (15-50 cm) soil horizons, with these horizons parametrized by depth according to Table S1. Fourteen different discretized depths were considered for the model space, with the near surface column being discretized in 0.5-cm and 1-cm increments to account for fluxes at the soil/air interface, and deeper portions of the column discretized in 5-cm increments. Figure S18 presents a schematic showing the discretization and boundary conditions of the 1D soil column simulation. During the draining phases of the experiments, the hydraulic boundary condition at the base of the soil column simulations was controlled with an input file of temporally changing outflow volume measured from the experimental soil columns. During the saturation phases, the bottom boundary condition was changed to a no-flow boundary. A spin-up phase was used to account for early-time variability in the model simulations.

**Table S1**. Parameters used for simulations of experimental columns.

^13-15^

To determine model sensitivity to physical and chemical soil properties, the saturated hydraulic conductivity (input as permeability), porosity, and the air (D_a_) and water (D_w_) diffusion coefficients were changed, and response variables were evaluated (Table S2). Liquid saturation, pH, Total aqueous Fe, Free DOM, Total aqueous acetate, Total bicarbonate, oxygen, and Total CH_4_ were used as response variables to the imposed changes. Input parameters for saturated hydraulic conductivity, K_sat_ (input as permeability in PFLOTRAN), were changed from the base case UC and TC simulations that were used to fit column experimental data. For K_sat_, the base case was increased or decreased an order of magnitude (10x K_sat_ or 0.1x K_sat_). Porosity was evaluated from 0.1 to 0.9 (min or max), and D_a_ and D_w_ were doubled or halved (2x D or 0.5x D).

**Table S2**. Parameters used for sensitivity analysis of PFLOTRAN model.

**Table S3.** Significant changes in relative abundances of bacterial families following water table manipulation experiments in the upland soils column


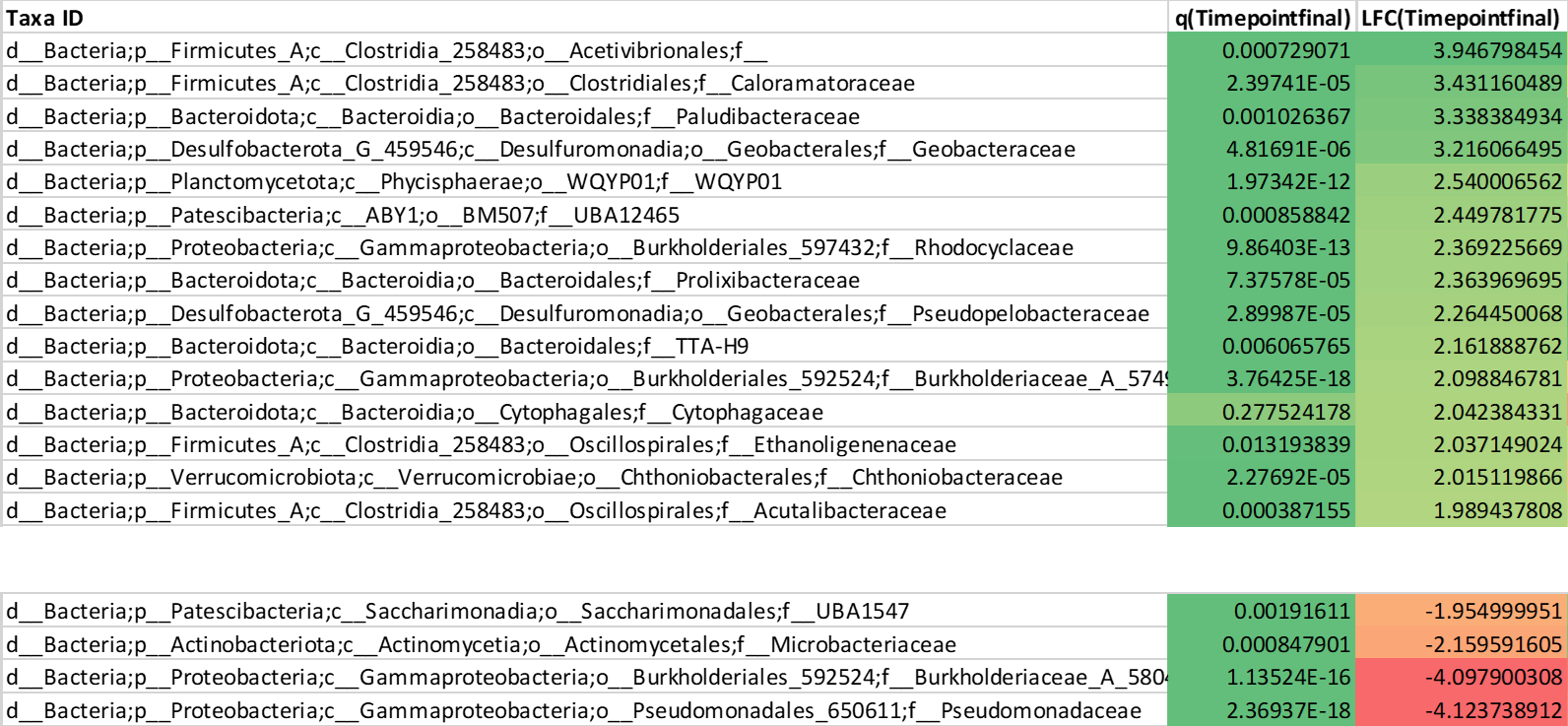
ANCOM-BC analysis identified greater than 3 log-fold increases in the abundance of *Acetivibrionales, Caloramatoraceae, Paludibacteraceae*, and *Geobacteraceae* families and greater than 4 log-fold decreases in *Burkholderiacaeae* and *Pseudomonadaceae* families after column incubations. Values are highlighted with colors corresponding to their relative ranks in a full list of taxa (green = high; red = low). LFC, Log-fold change.

**Table S4**. Average iron (Fe) oxidation states derived from linear combination fits to the XANES regions. Iron in organic soils shows evidence for being more oxidized than mineral soils, and both organic and mineral soils showed slight increases in average Fe oxidation state post-experiment.

| **Data** | **chinu** | **Fe(II)** | **Fe(III)** | **Fe(avg)** |
| --- | --- | --- | --- | --- |
| EB01-UC-pre-org | 0.000952 | 0.26 | 0.74 | 2.74 |
| EB03-TC-pre-org | 0.000529 | 0.37 | 0.63 | 2.63 |
| EB05-TC-post-org | 0.000522 | 0.29 | 0.71 | 2.71 |
| EB02-UC-pre-min | 0.000502 | 0.73 | 0.27 | 2.27 |
| EB04-TC-pre-min | 0.000142 | 0.81 | 0.19 | 2.19 |
| EB06-TC-post-min | 0.000573 | 0.63 | 0.37 | 2.37 |

**Table S5**. Iron (Fe) components determined from linear combination fits to the EXAFS region for the organic soils in both experiments.

| Sample Name | No. Fits | Red.  Chi.Sq. | Fh (%) | ± | Org-Fe(III) (%)^b^ | ± | Silicate-Fe (%)^c^ | ± | Other Fe(II) (%)^d^ | ± |
| --- | --- | --- | --- | --- | --- | --- | --- | --- | --- | --- |
| EB03-TC-pre-org | 3 | 0.020 | 66.7 | 2.9 | 18.5 | 11.3 | 6.6 | 4.3 | 8.2 | 0.3 |
| EB04-TC-pre-min | 1 | 0.013 | 29.2 | 3.6 | 19.0 | 3.2 | 51.8 | 9.0 | 0.0 | 0.0 |
| EB02-UC-pre-min | 3 | 0.022 | 44.2 | 3.3 | 18.8 | 15.4 | 25.8 | 1.0 | 11.2 | 0.4 |
| EB01-UC-pre-org | 1 | 0.007 | 31.3 | 2.7 | 50.5 | 2.5 | 13.1 | 1.2 | 5.0 | 4.4 |
| EB05-TC-post-org | 1 | 0.009 | 51.6 | 3.0 | 31.5 | 2.8 | 10.0 | 1.4 | 7.0 | 5.2 |
| EB06-TC-post-min | 1 | 0.006 | 27.4 | 2.4 | 29.2 | 2.2 | 39.8 | 1.1 | 3.5 | 3.7 |

| ^a^Error (±) is the mean standard error of multiple fits or the error reported with the single fit |
| --- |
| ^b^Organic-Fe(III) is the sum of Fe(III)-citrate and Fe(III)-oxalate |
| ^c^Silicate-Fe is the sum of biotite and chlorite |
| ^d^Other Fe(II) is represented in the fits as pyrite |

Ferrihydrite and Fe(III)-citrate indicate different oxidized iron phases and pyrite likely represents different types of Fe(II) reduced iron phases including reduced iron phases that could be sulfides, siderite or other phases. The upland column (UC) organic soil indicates higher Fe(III)-citrate than the TC organic soils. Pre and post experiment TC data shows more oxidation of iron with increases in ferrihydrite and Fe(III)-citrate post experiment.

# S2. Supporting Figures

**
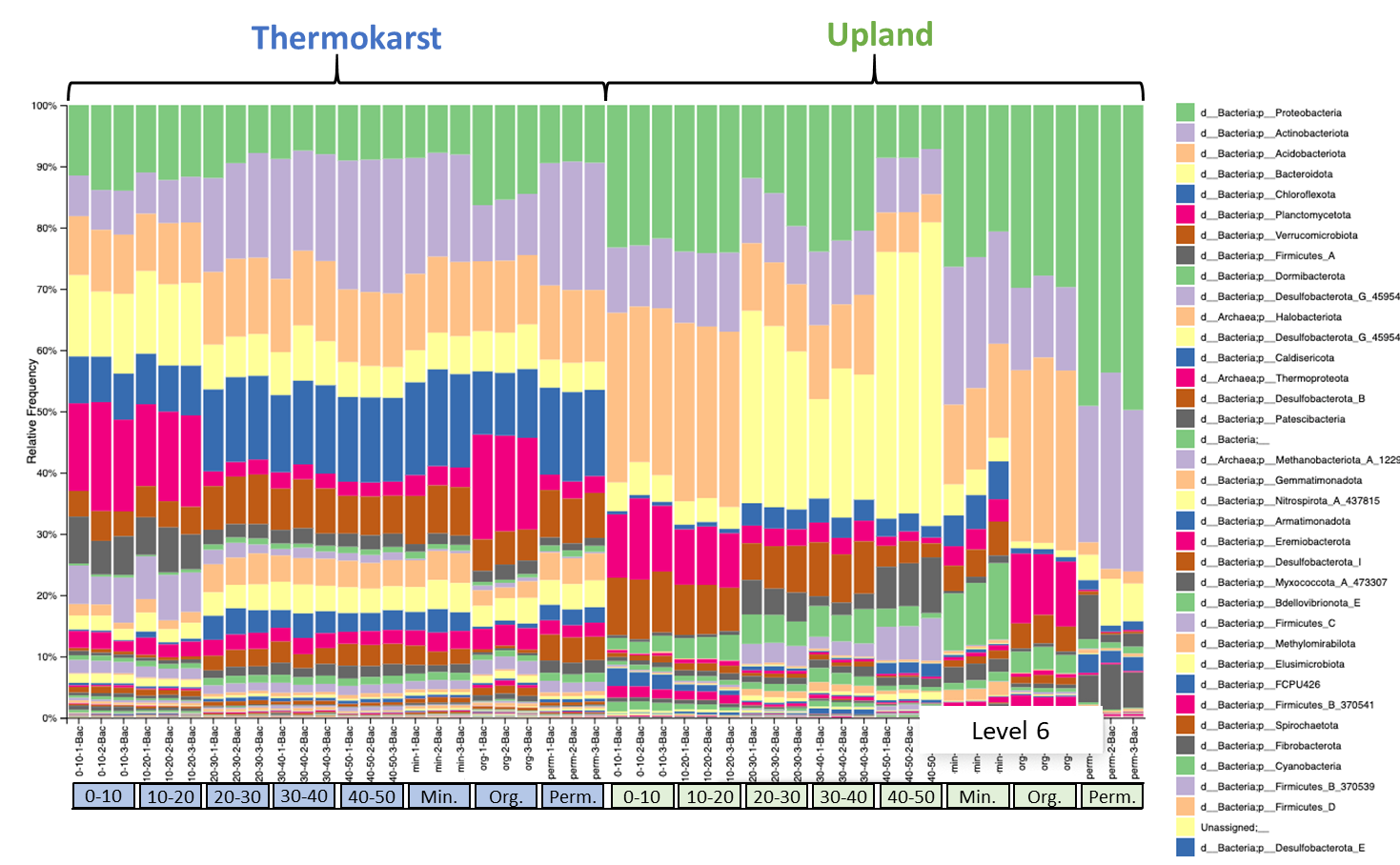
**

**Figure S1.** Phylum-level taxonomic classification of bacterial and archaeal compositions using the Greengenes 2 database identified distinct community structures in thermokarst (TC) and upland (UC) column soils, as well as depth-dependent differences. Pre-experiment samples are indicated with Org., Min., and Perm. for organic, mineral, and permafrost/deep samples, respectively. Post-experiment samples include 0-10 cm and 10-20 cm depths for organic soils, 20-30 cm and 30-40 cm for mineral soils, and 20-50 cm for permafrost in upland or deep soils in thermokarst experiments.

**
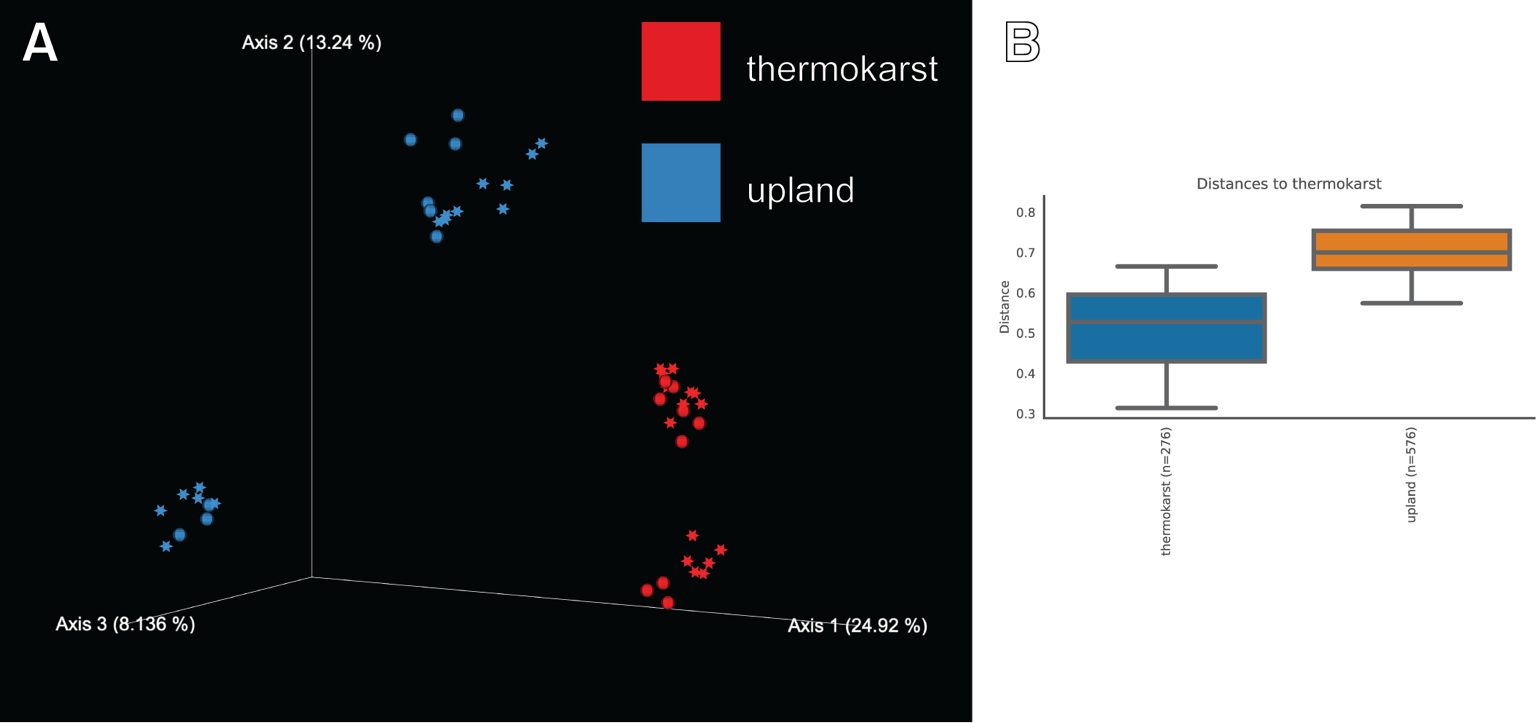
**

**Figure S2**. β-diversity of microbial communities was primarily due to topographic soil position. Emperor plot (S1A) of unweighted UniFract distance metrics identified distinct microbial communities from upland (blue) or thermokarst (red) soils. Spheres indicate initial communities, while stars indicate final communities. PERMANOVA analysis (S1B) identified a significant difference in distances (p-value = 0.001).

**
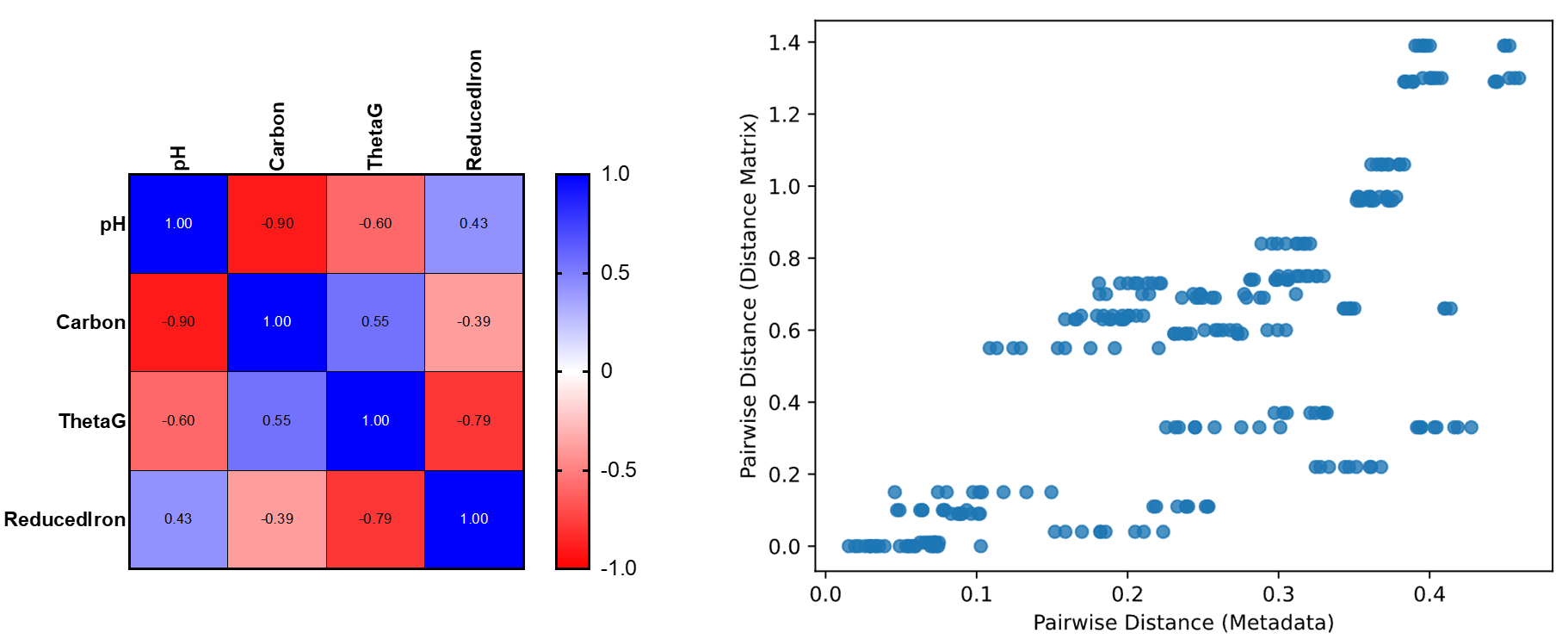
**

**Figure S3**. For upland soils, pH, soil layer, and timepoint were the best predictors of microbial community β-diversity. Left, a metadata correlation matrix illustrates strong negative correlations between upland soil pH and carbon, and between gravimetric soil water content (ThetaG, θ_g_) and reduced iron (Fe(II)) content. Right, a multifactor PERMANOVA plot (Adonis) shows pH strongly predicts β-diversity.

**
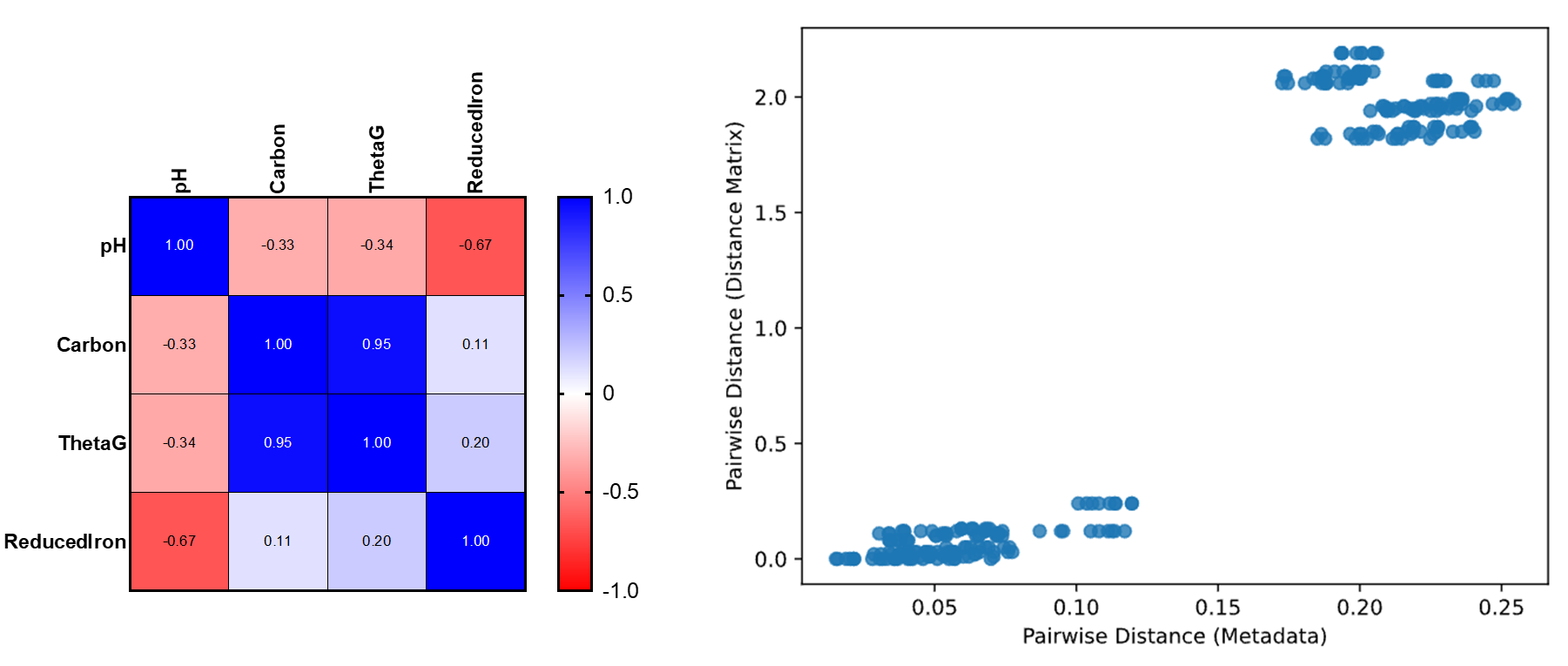
**

**Figure S4.** For thermokarst soils, water content (ThetaG, θ_g_) and carbon were the best predictors of microbial community β-diversity. Left, a metadata correlation matrix illustrates strong positive correlations between thermokarst soil water content (ThetaG, θ_g_) and carbon, and a negative correlation between pH and Fe(II) content. Right, a multifactor PERMANOVA plot (Adonis) shows θ_g_ strongly predicts β-diversity.


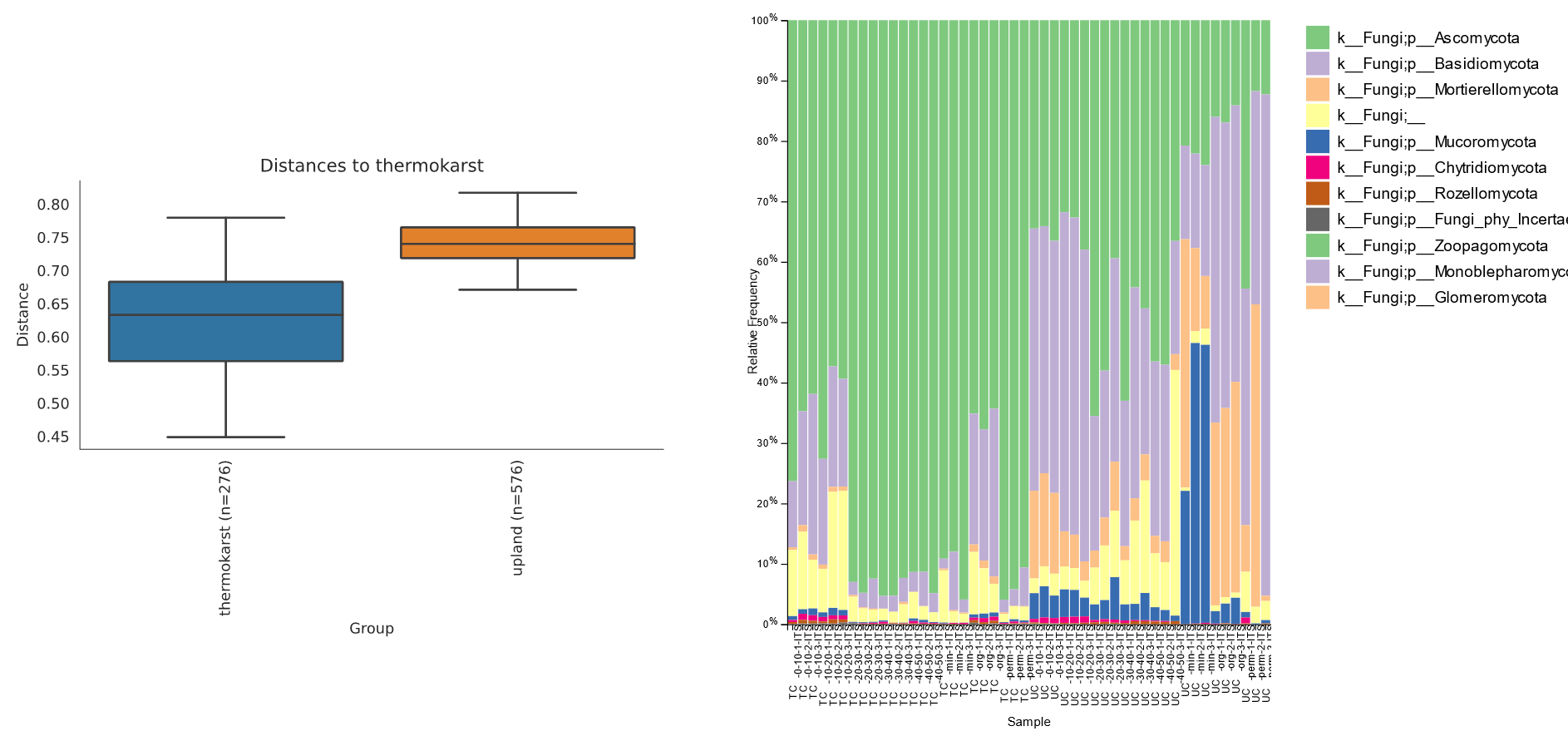


**Figure S5.** PERMANOVA analysis identified a significant difference in distances (p-value = 0.001) between communities in the thermokarst and upland soils. Right, Phylum-level taxonomic classification of fungal compositions using the UNITE QIIME 2 database identified distinct community structures in thermokarst (TC) and upland (UC) column soils, as well as depth-dependent differences.


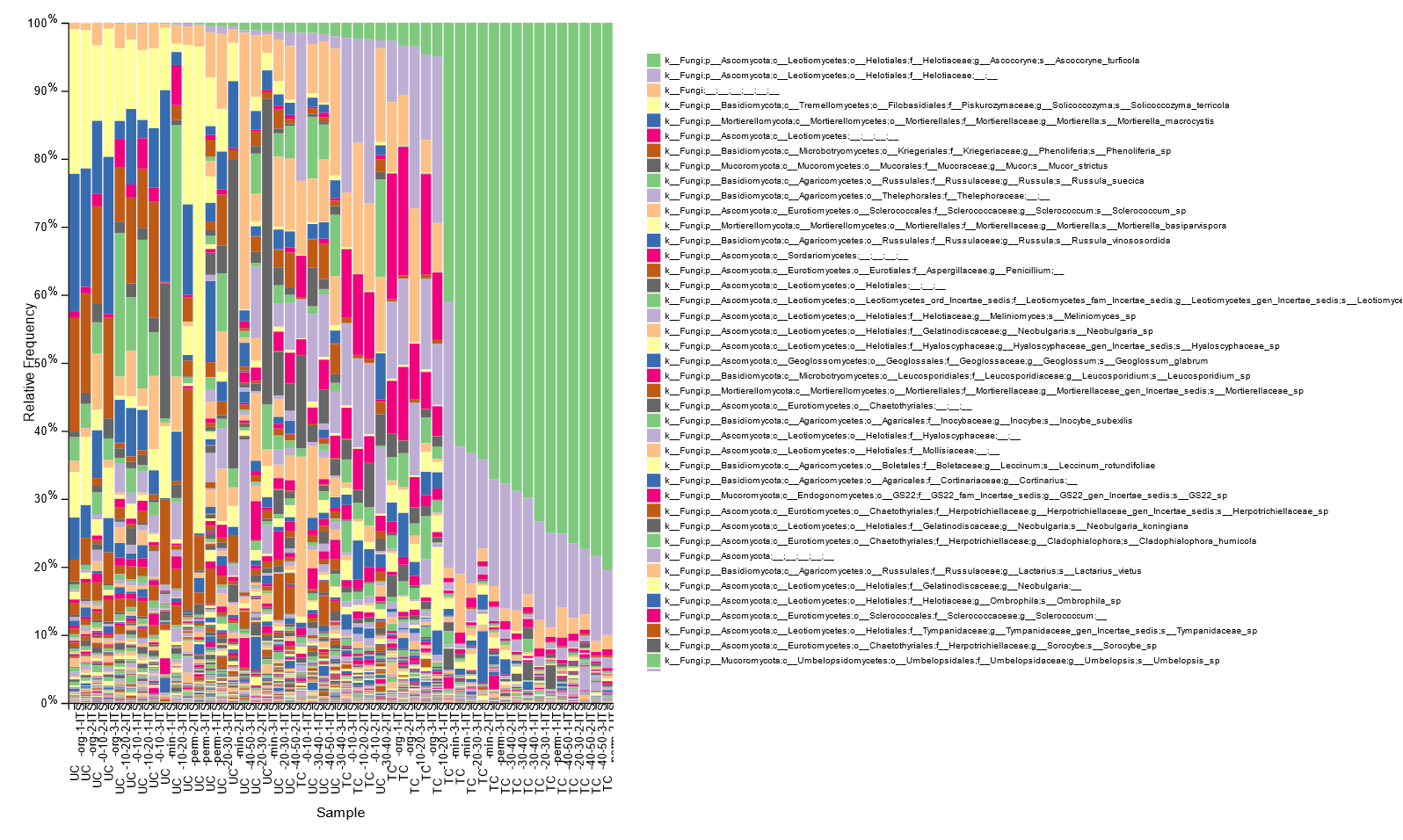


**Figure S6.** Species-level taxonomic classification of fungal compositions identified *Ascocoryne turficola* as the dominant species in deep thermokarst (TC) soils.


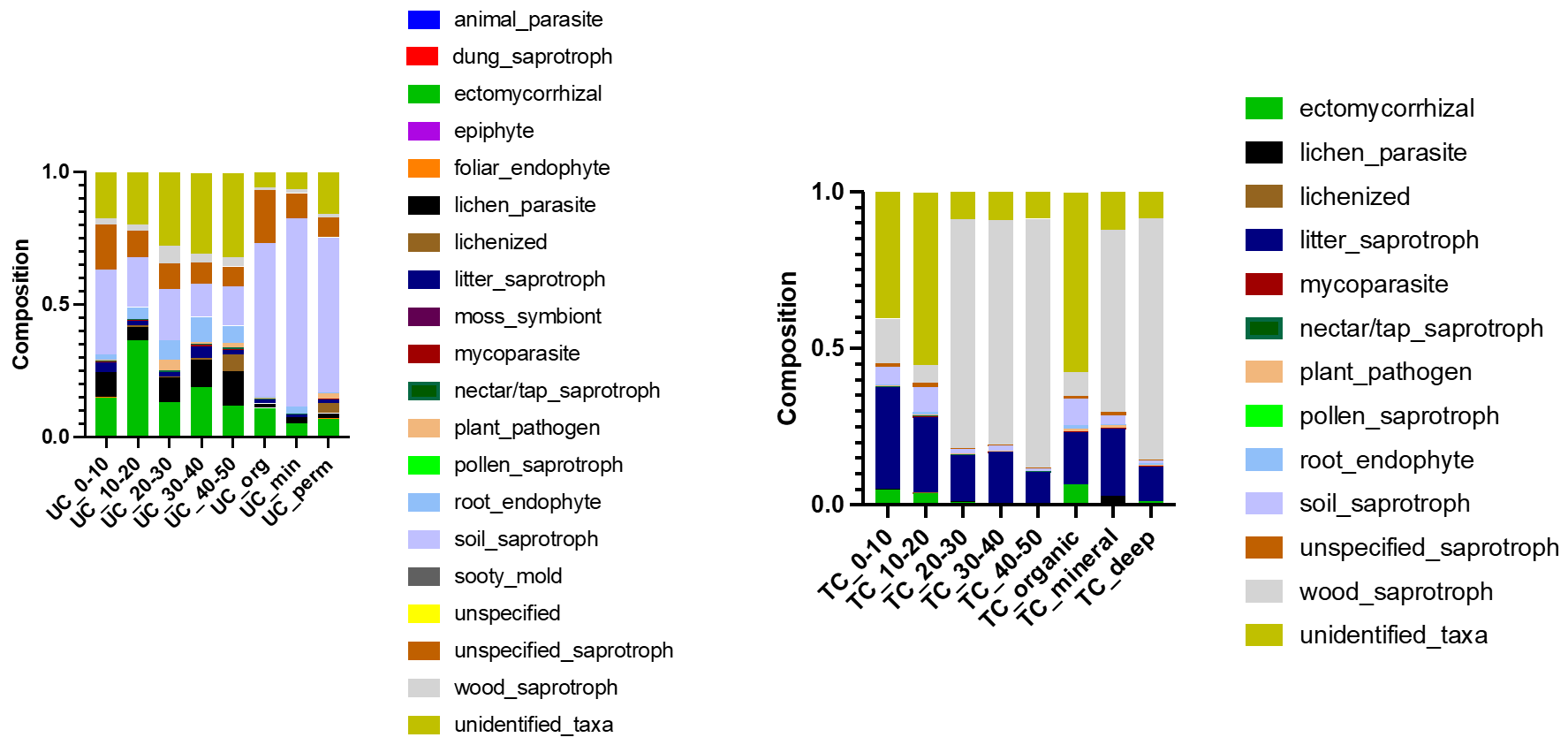


**Figure S7.** Ecological functions of fungal community composition predicted from the FungalTraits database for upland (UC, left) and thermokarst (TC, right) soils in the indicated layer

**
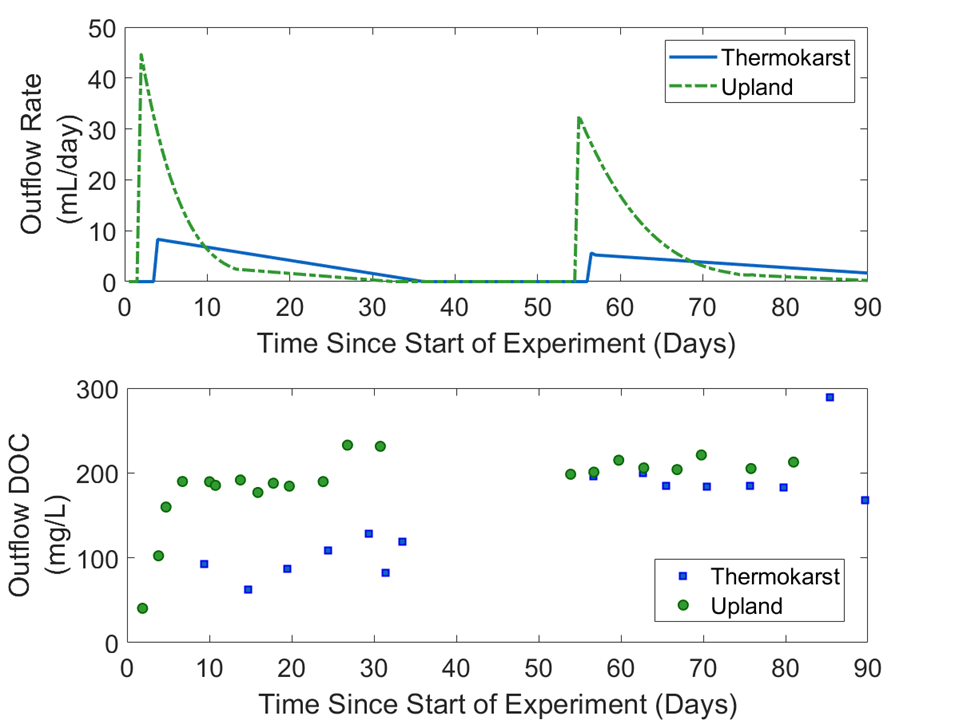
**

**Figure S8**. Outflow timeseries data represented as an outflow rate (integrating outflow volume from Figure 2A in the main article) and the outflow dissolved organic carbon (DOC) concentration for both upland and thermokarst column experiments. The outflow rates were used as inputs for PFLOTRAN simulations to quantify drainage rates.


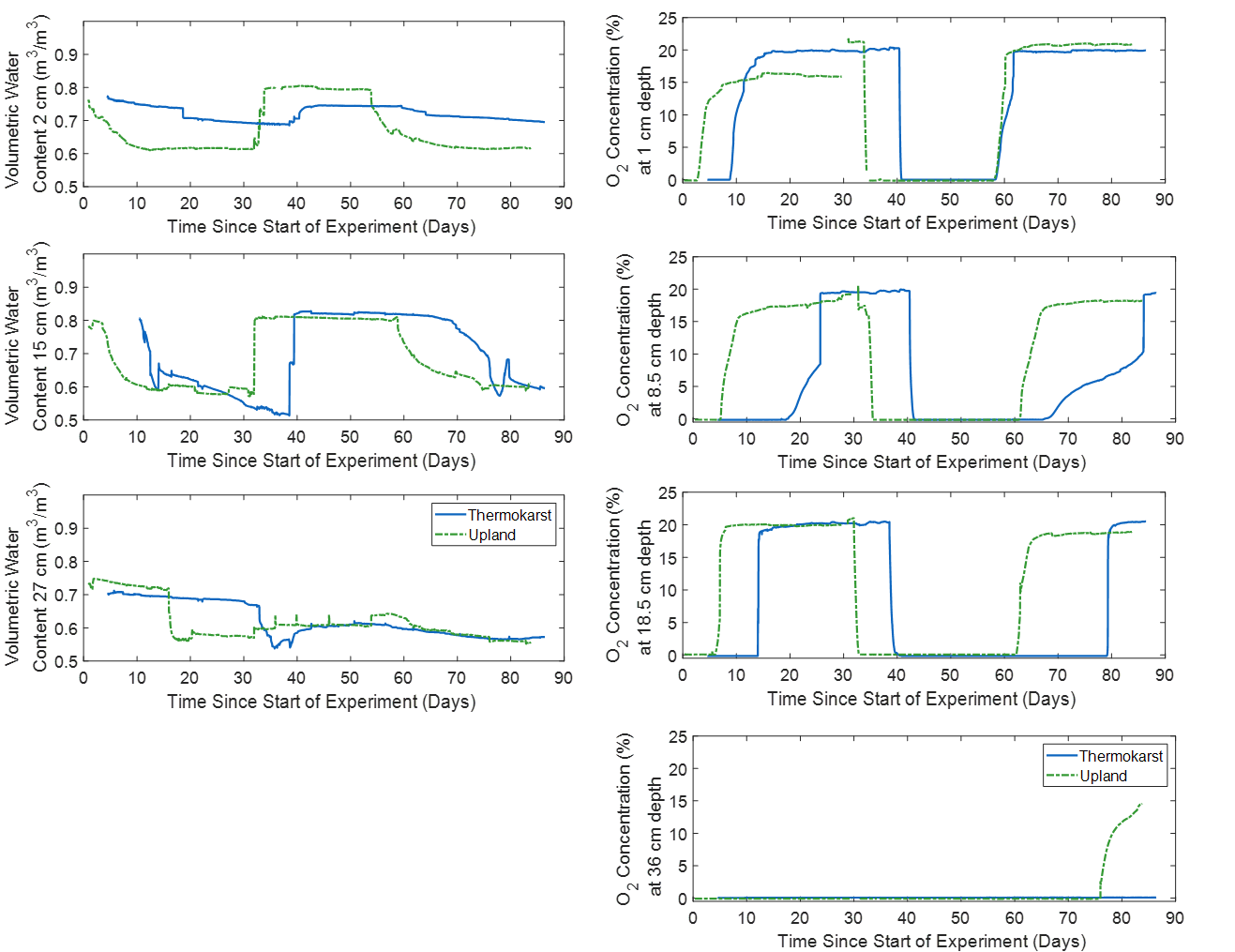


**Figure S9**. Temporal changes in volumetric water content (VWC) for three sensor depths and oxygen (O_2_) for four sensor depths. Vertical axes indicate depth of sensors in soil column. Green, dashed series represents the upland column, and the blue line represents the thermokarst column. The gaps and steps in the O_2_ concentrations for 1 cm depth are associated with power outages and a removal of the oxygen sensor for recalibration; other depths were less impacted by recalibration than the 1 cm depth. VWC for the sensors appeared to be higher than measurements in the field (Figure S4), likely associated with a lack of plant-mediated evapotranspiration.


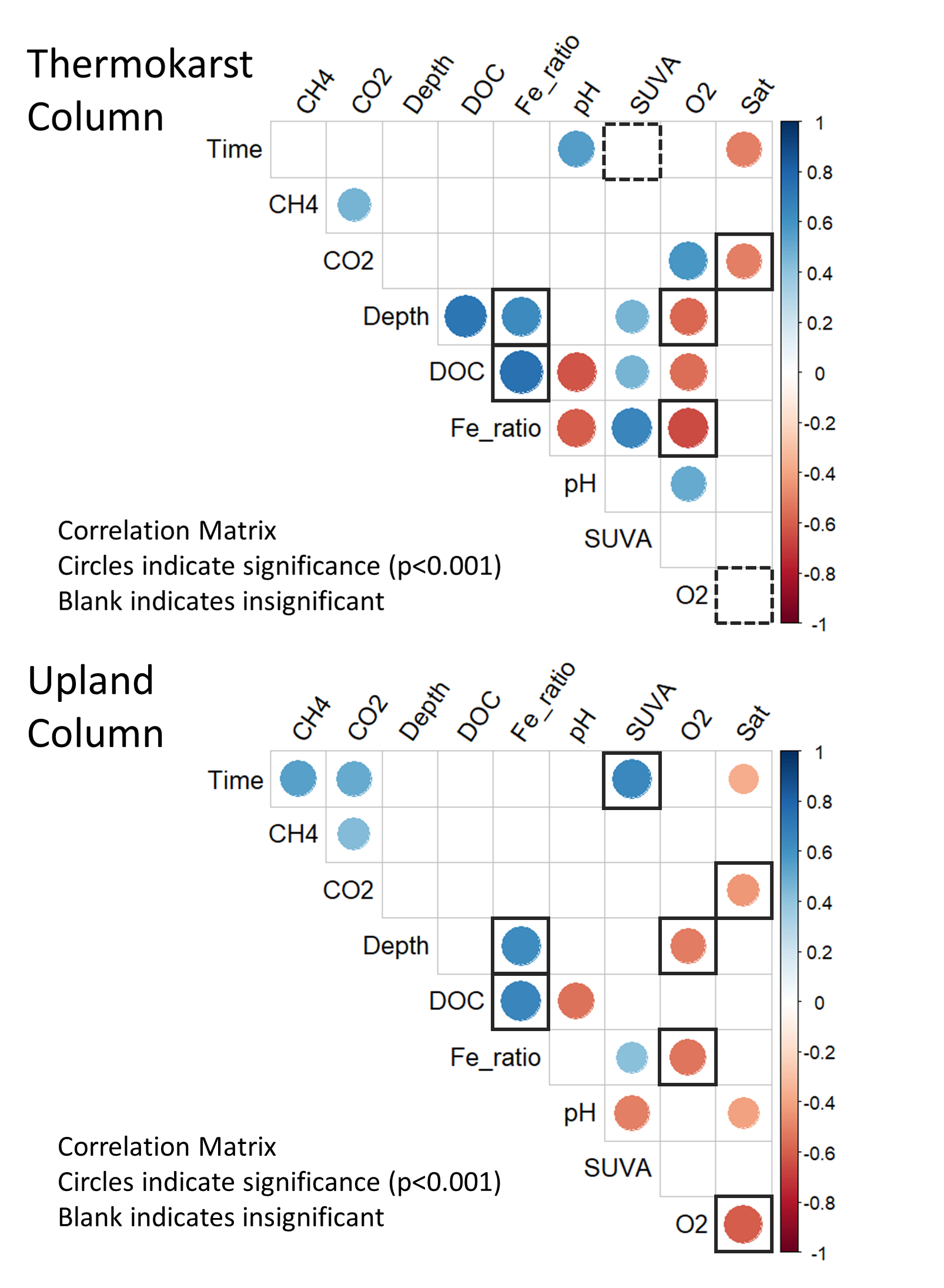


**Figure S10**. Correlation matrices for thermokarst and upland column measurements. The presence of a circle indicates a correlation with p<0.001, and the size and color of the circle is related to the extent of positive or negative correlation (also represented in the color bar). Black boxes for certain correlations aid in comparing major trends between upland and thermokarst column experiments. For example, correlations for SUVA and time or saturation and O_2_ did not show strong correlations for the thermokarst column experiment but did show correlations for the upland column experiment.

**Figure S11**. Timeseries data for dissolved organic carbon (DOC), pH, and Ferrous:Total iron at all five port depths for both upland and thermokarst column experiments.


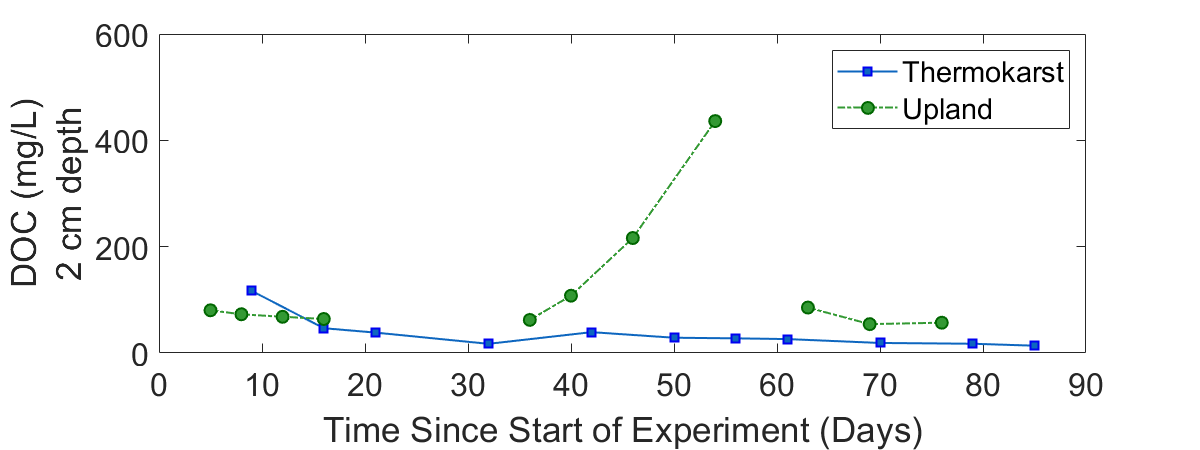

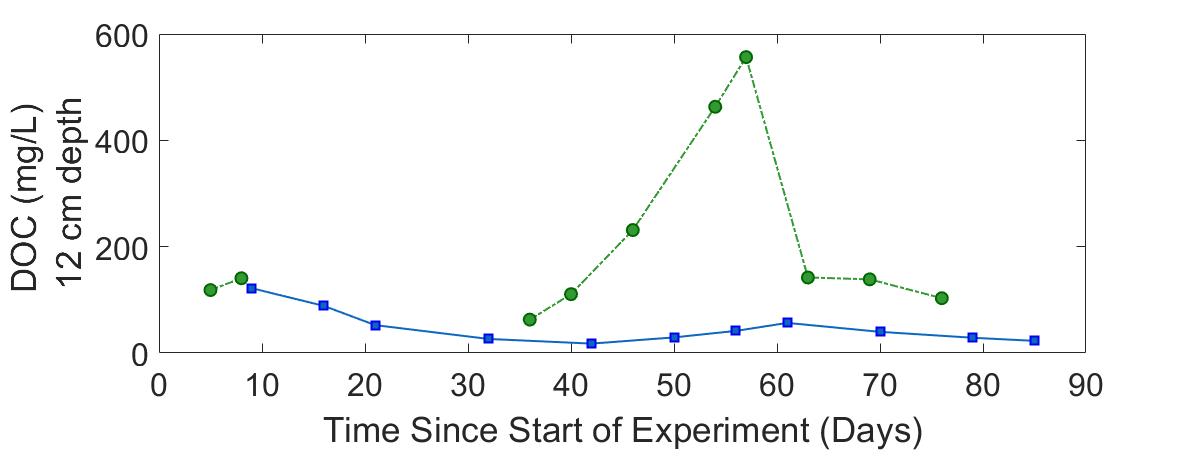

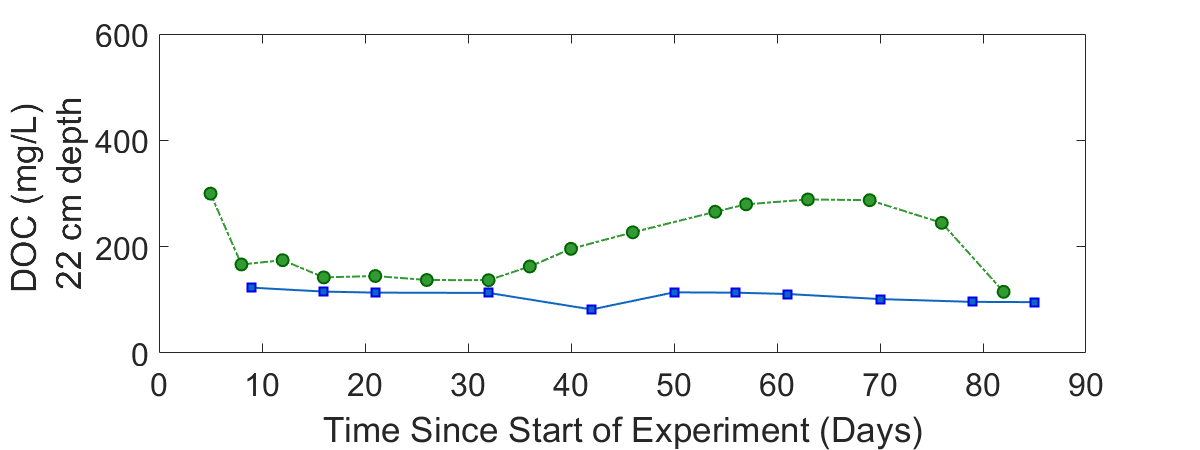

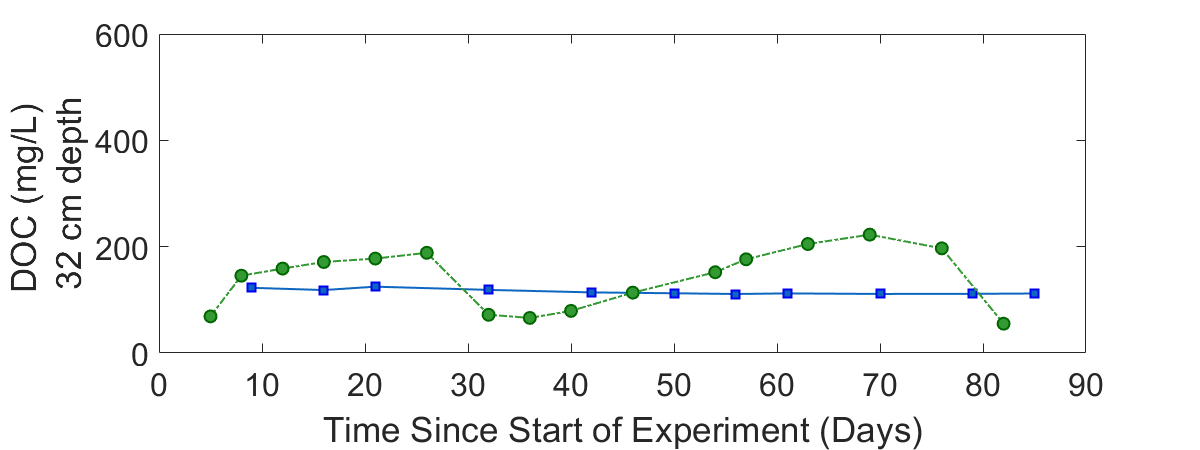

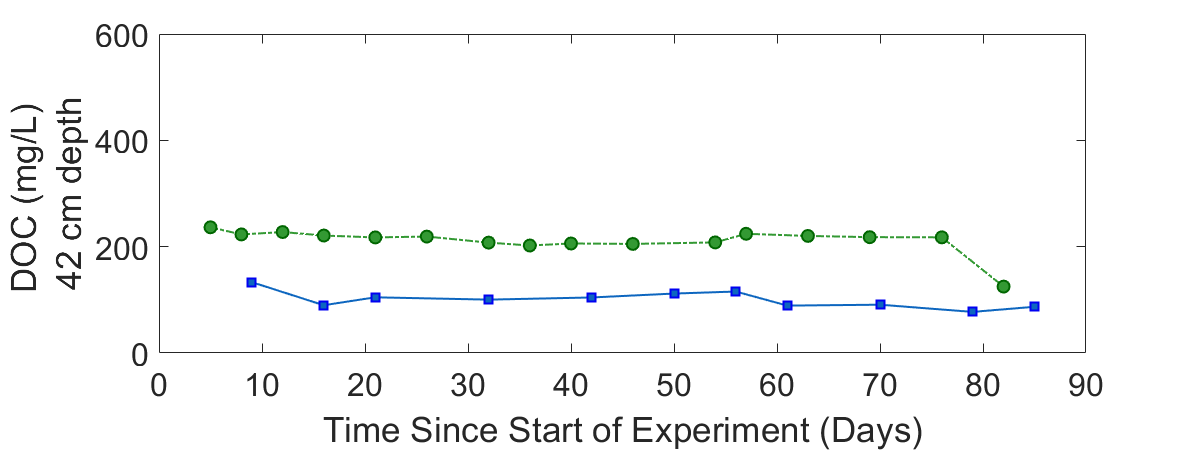

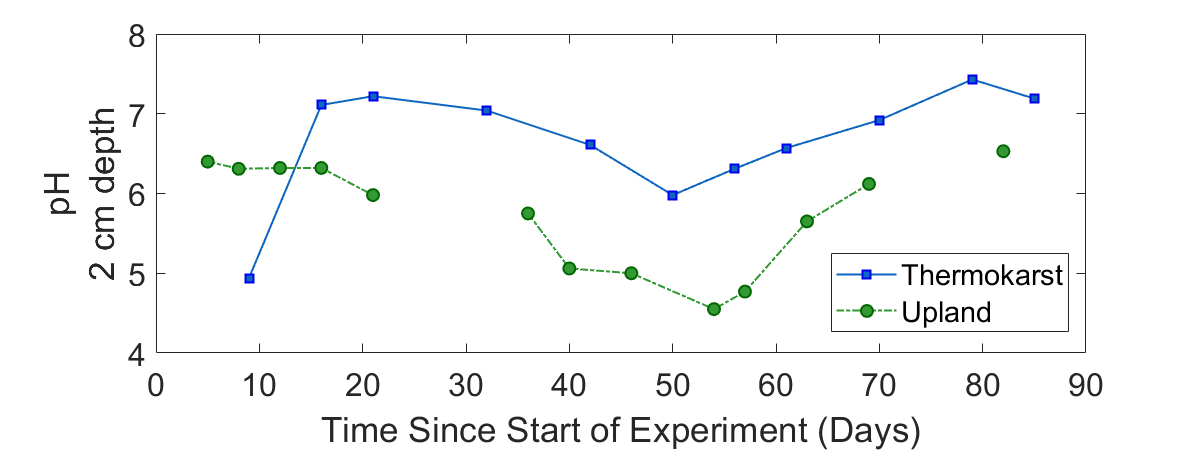

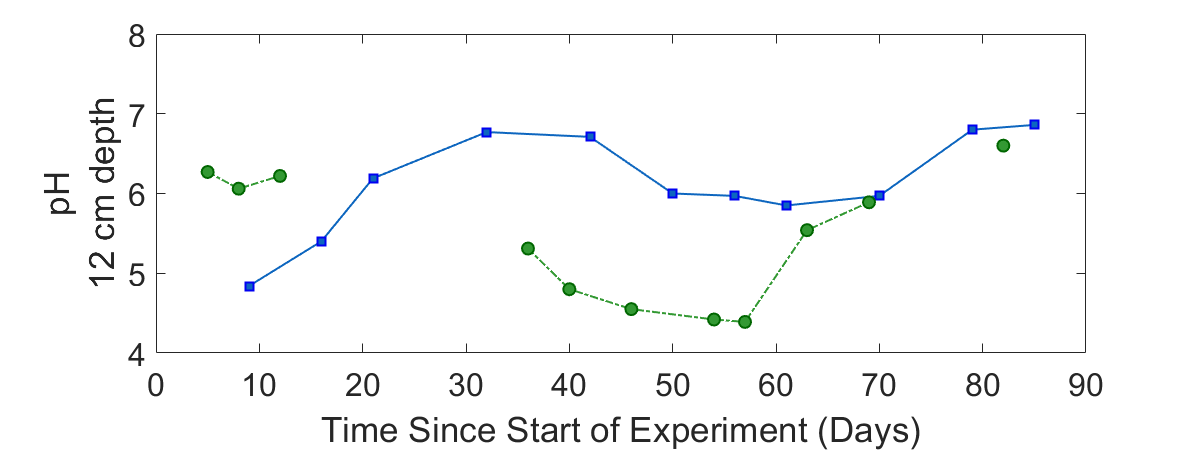

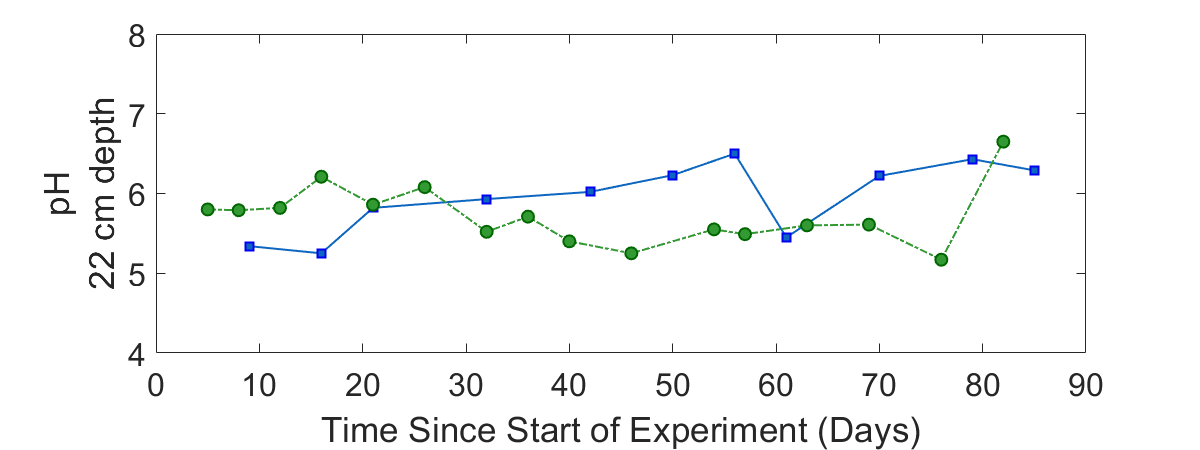

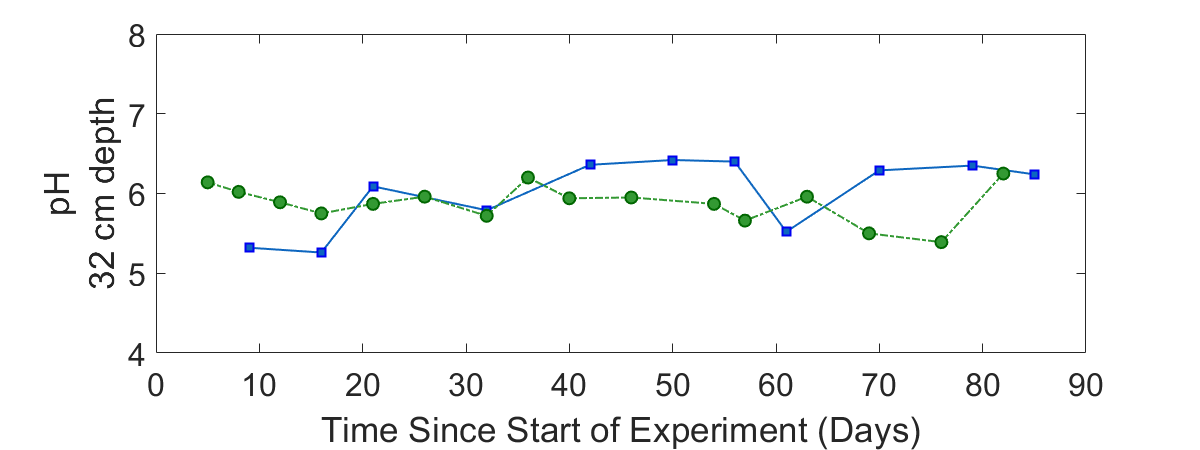

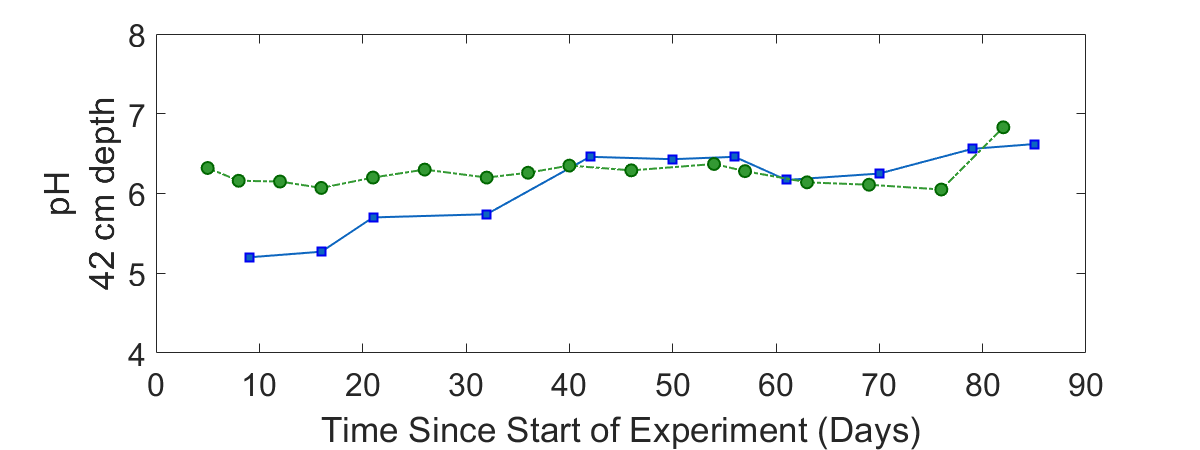

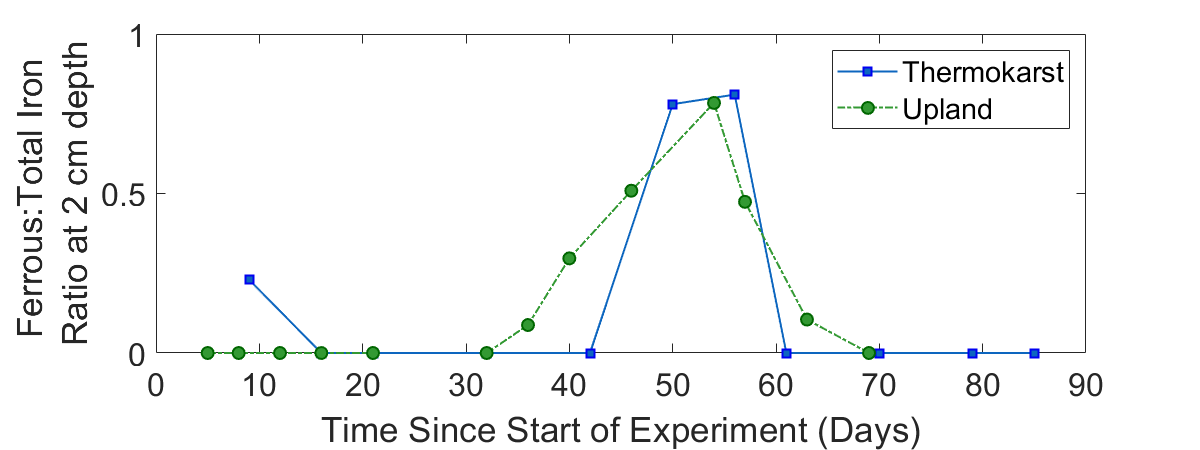

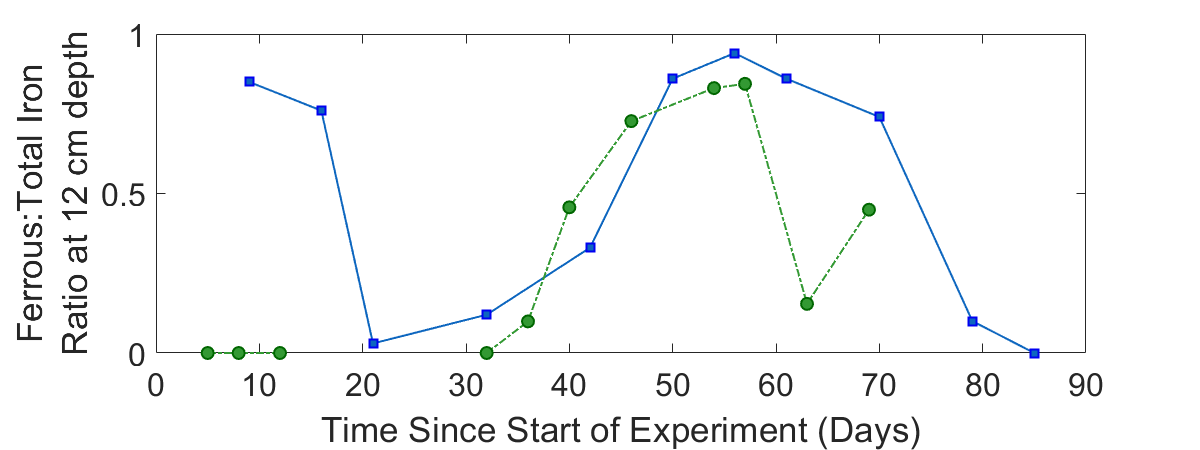

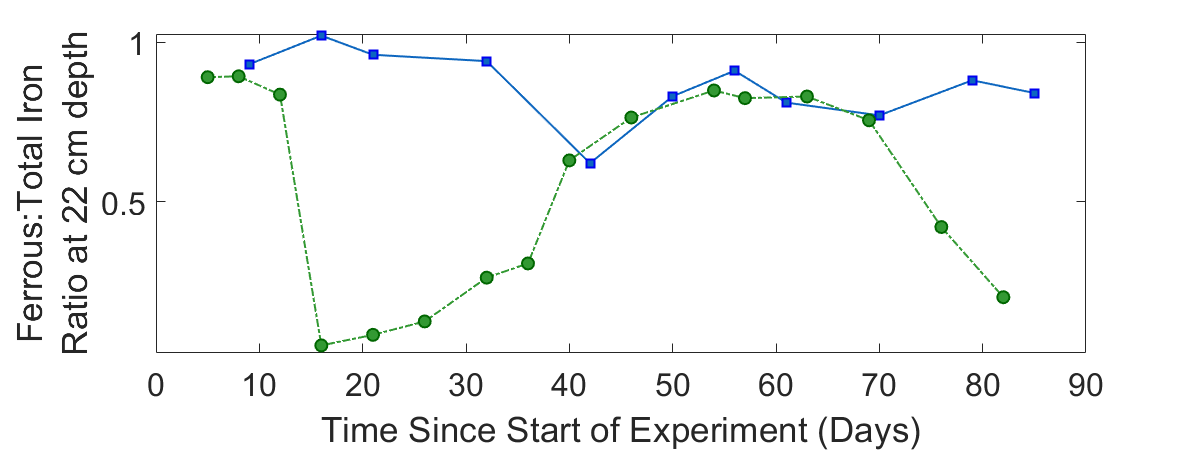

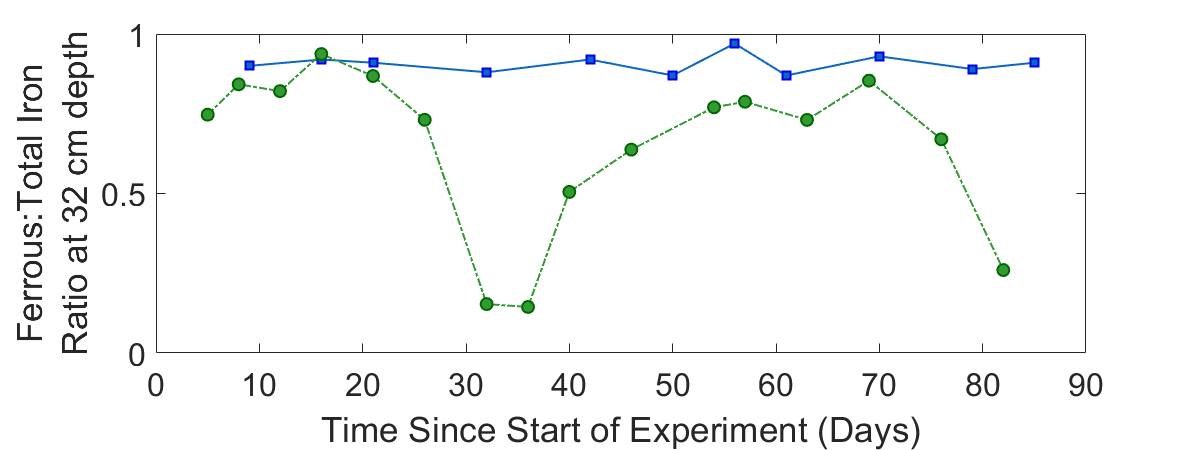

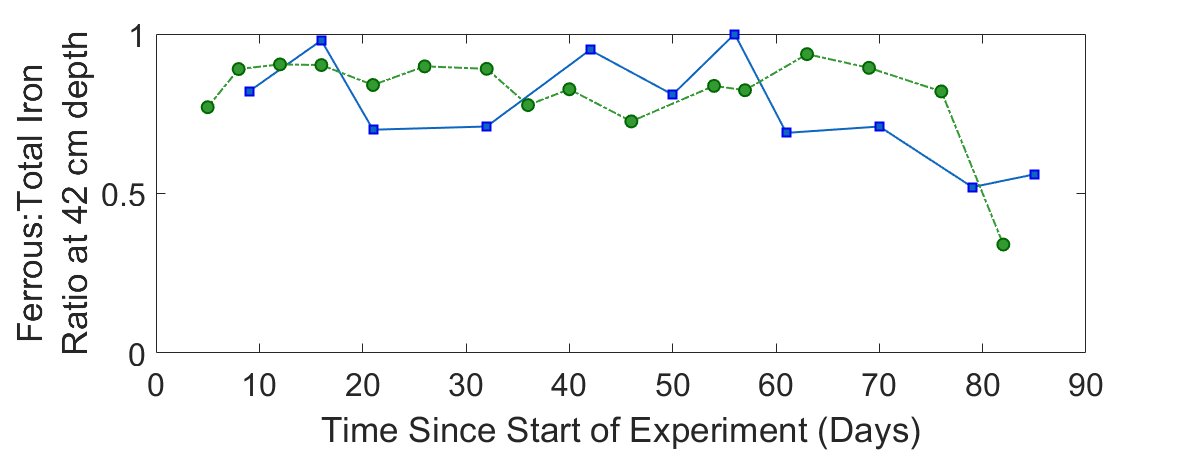


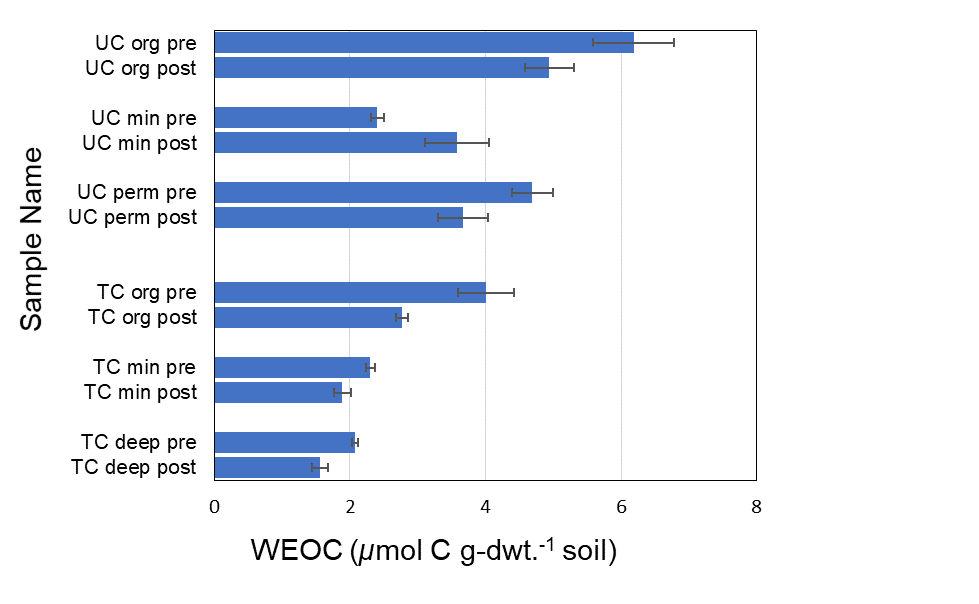


**Figure S12**. Water extractable organic carbon (WEOC) for pre and post experiment soil samples. The upland column (UC) showed an increase in WEOC in the mineral soil, possibly indicating transport from organic soil down to the mineral soil, whereas all other soils in both experiments (UC and TC) showed a decrease in WEOC from pre to post experiment. These trends mirror the iron redistribution in the UC column shown in Figure 4 (see main text).

**Figure S13**. Iron K-edge spectra showing differences in iron speciation between mineral (blue) and organic (green) soils. Post-experiment soils for the thermokarst column experiment (TC) are shown as dashed lines. Pre-experiment for both upland (UC) and TC experiments are solid lines.

**
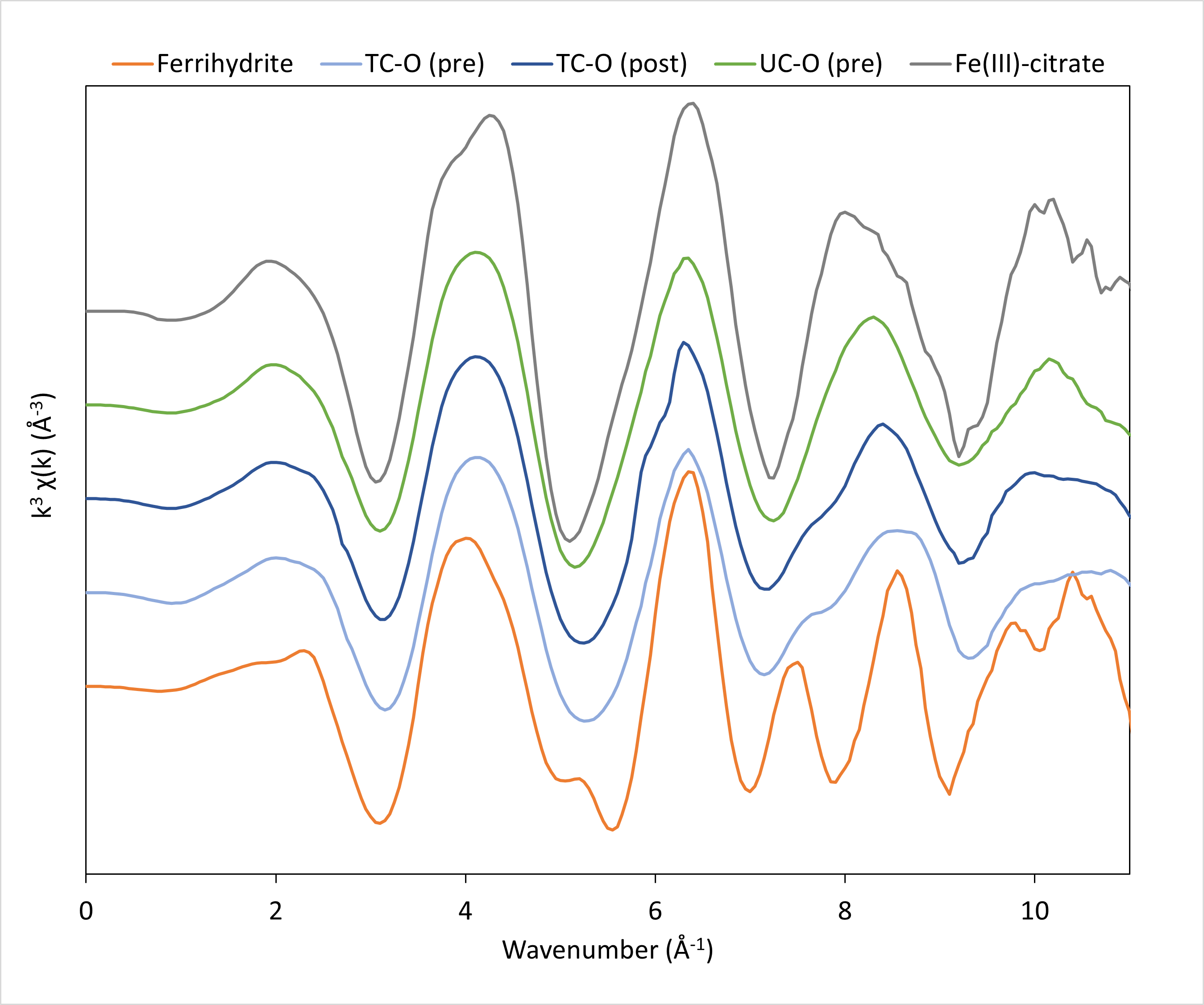
**

**Figure S14**. EXAFS region for upland (UC) and thermokarst (TC) organic soils. Purple series is a 6-line ferrihydrite standard and the yellow-green series is the iron(III)-citrate standard. UC organic soils show a stronger signature for iron(III)-citrate, as indicated by the single peak near 8 Å^-1^, and TC organic soils show a stronger signature for 6-line ferrihydrite as evidenced by the double peak near 8 Å ^-1^.


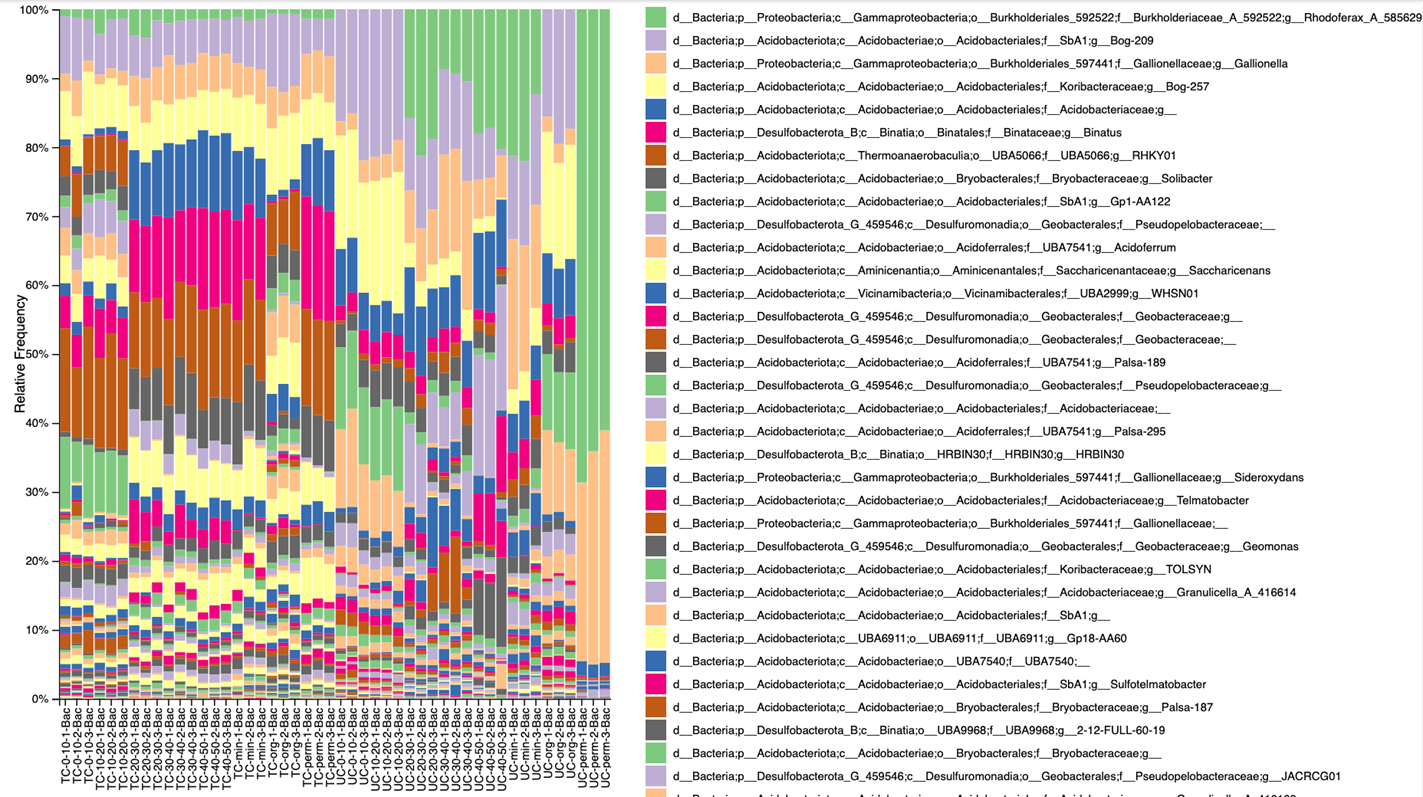


**Figure S15.** Compositions of potential iron-active bacteria at the genus level varied between thermokarst (TC) and upland (UC) soils and permafrost.

**
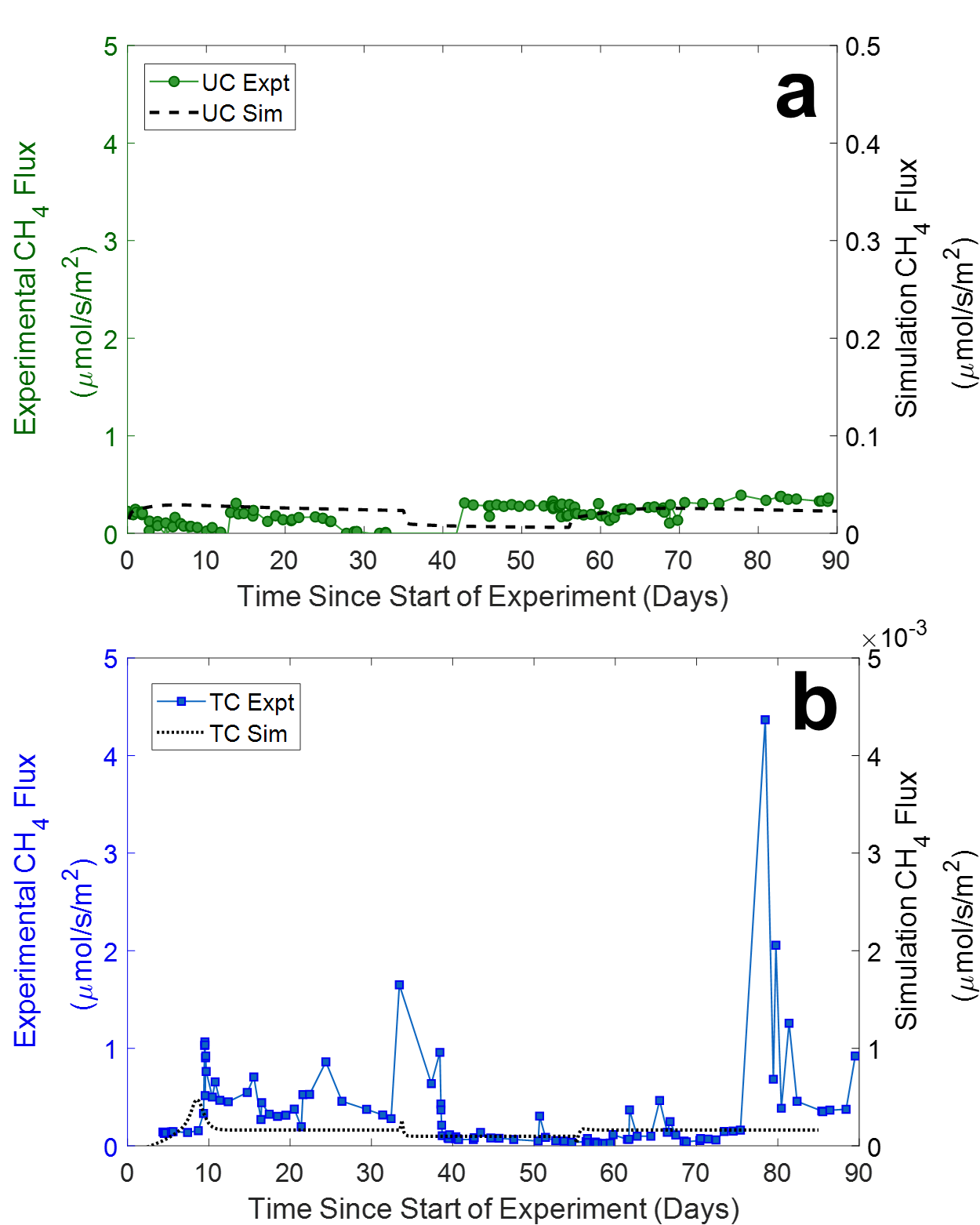
**

**Figure S16**. PFLOTRAN simulations compared to column experiment data for the upland column (UC, part a) and thermokarst column (TC, part b). Headspace CH_4_ fluxes from the experiment (colored markers) are plotted with PFLOTRAN simulation results (black dashed lines).


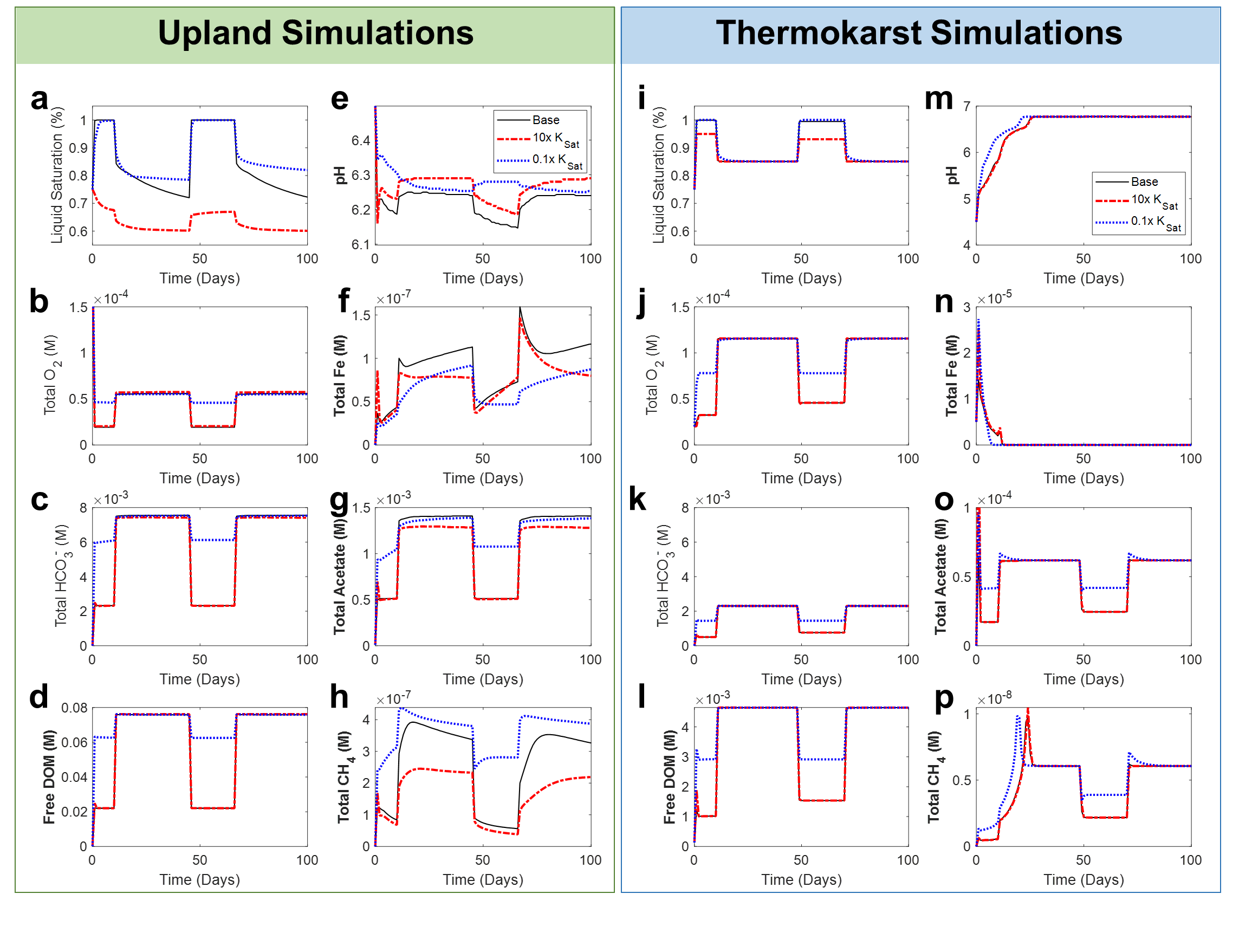


**Figure S17.** Summary of response variable changes due to sensitivity analysis with saturated hydraulic conductivity (K_sat_) of PFLOTRAN model. Selected response variables include liquid saturation, oxygen, pH, dissolved bicarbonate, total iron (Fe) dissolved oxygen (O_2_), free DOM, total CH_4_, and total acetate concentration, expanding results from Figure 6. Vertical axes in bold indicate that upland (left) and thermokarst (right) subplots do not have the same vertical axes.

**
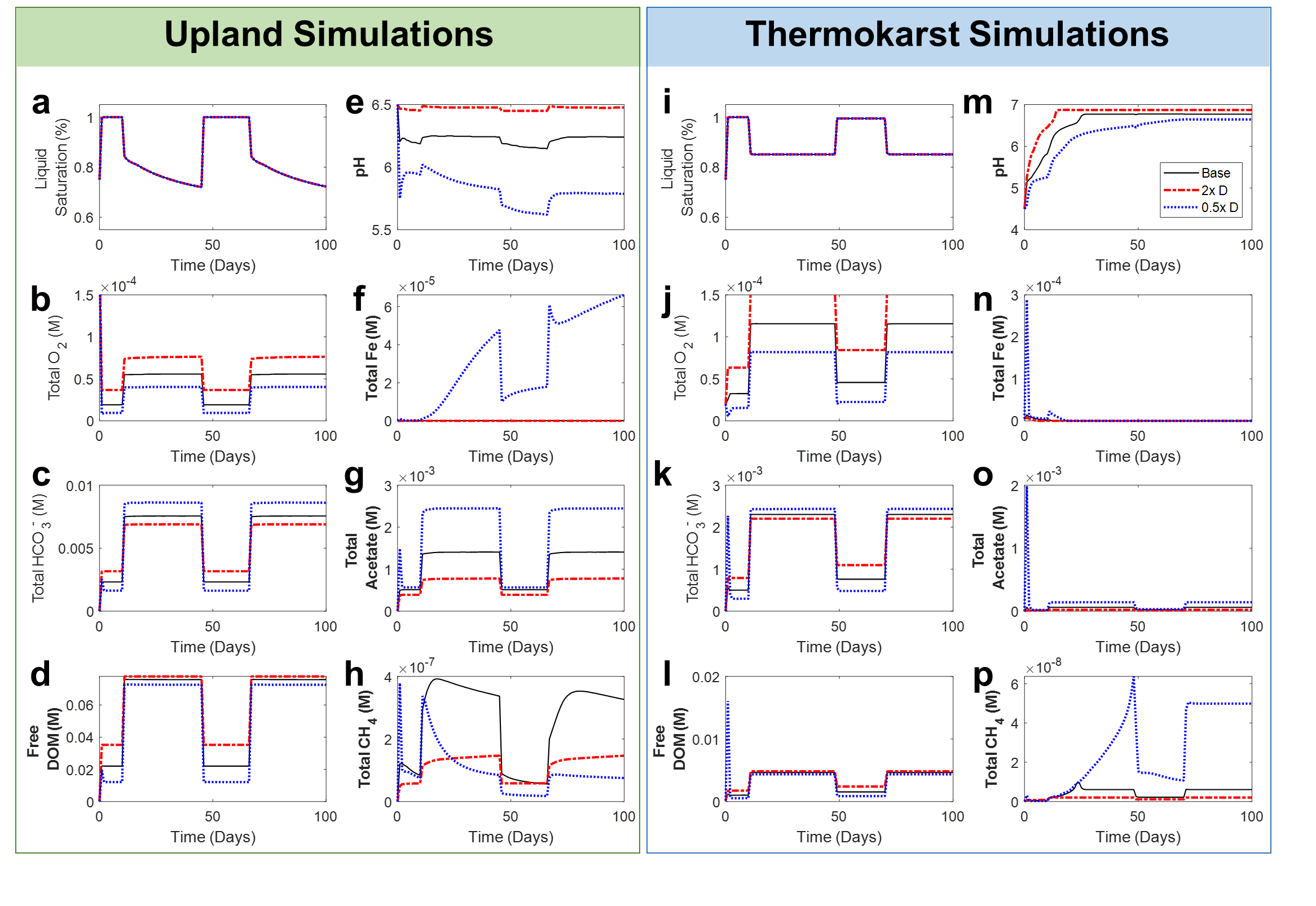
**

**Figure S18.** Summary of response variable changes due to sensitivity analysis with air and water diffusion coefficients (D_a_ and D_w_) of PFLOTRAN model. Selected response variables include liquid saturation, oxygen, pH, dissolved bicarbonate, total iron (Fe) dissolved oxygen (O_2_), free DOM, total CH_4_, and total acetate concentration. Vertical axes in bold indicate that upland (left) and thermokarst (right) subplots do not have the same vertical axes.


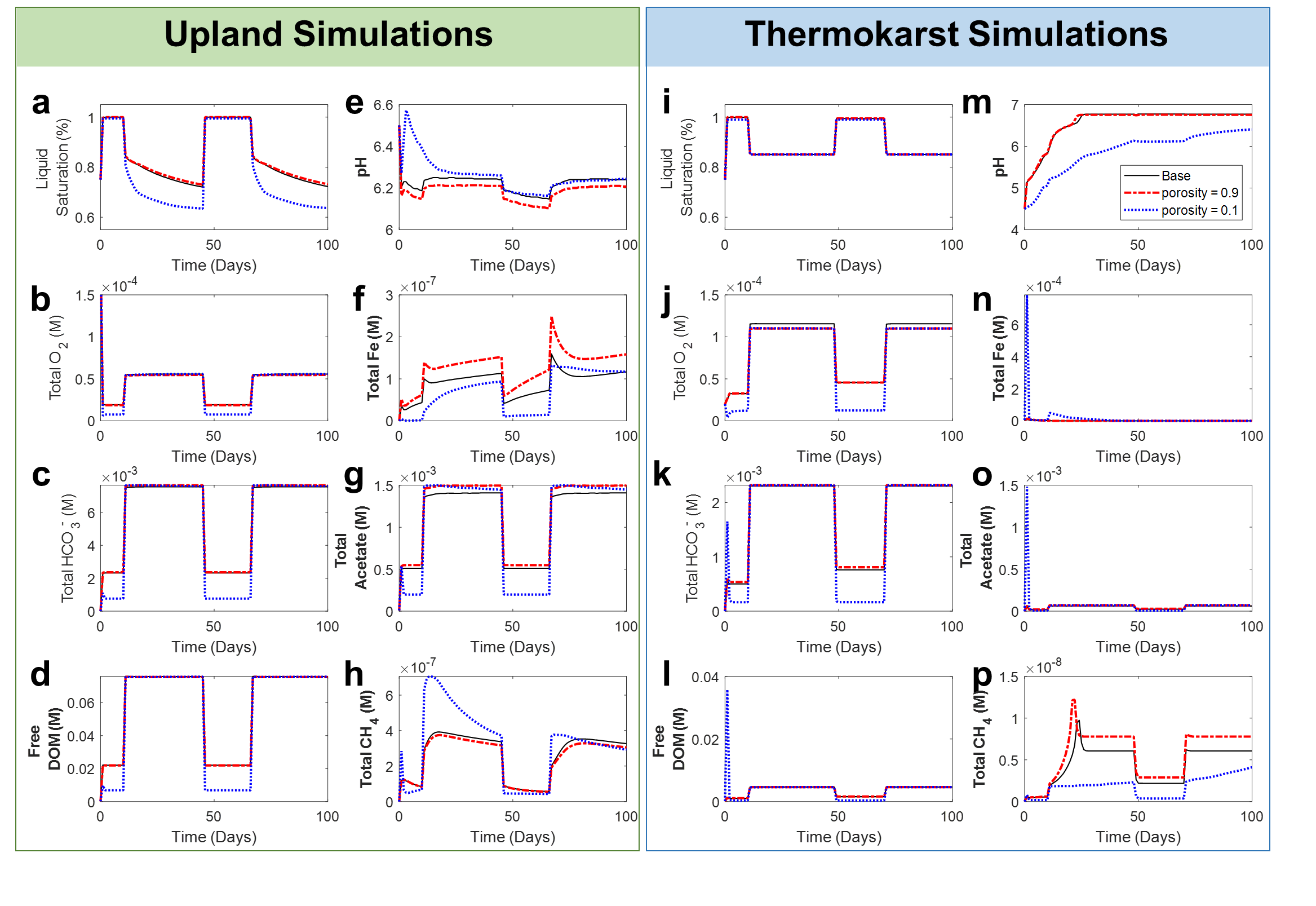


**Figure S19.** Summary of response variable changes due to sensitivity analysis with porosity (n) of PFLOTRAN model. Selected response variables include liquid saturation, oxygen, pH, dissolved bicarbonate, total iron (Fe) dissolved oxygen (O_2_), free DOM, total CH_4_, and total acetate concentration. Vertical axes in bold indicate that upland (left) and thermokarst (right) subplots do not have the same vertical axes.


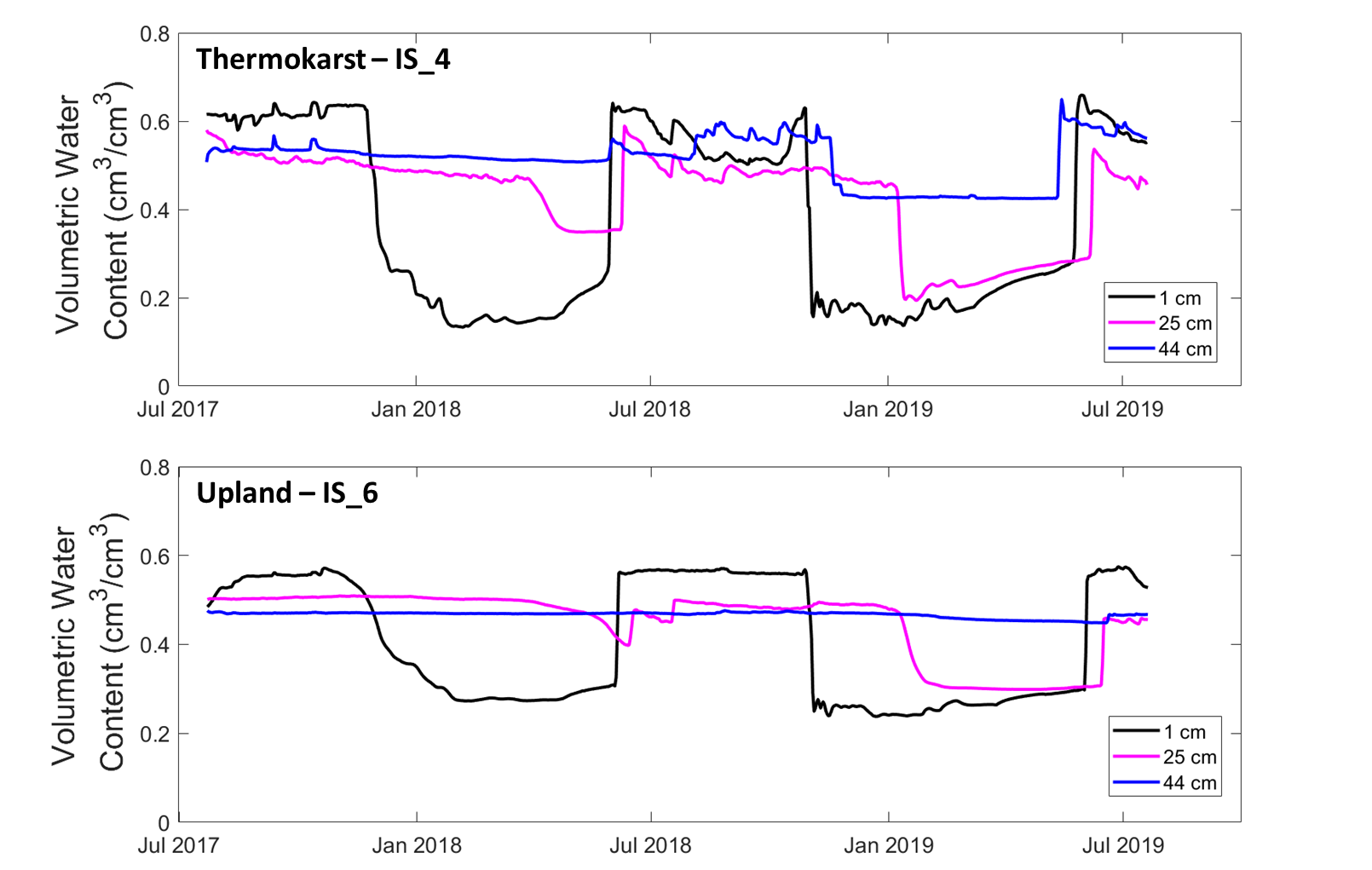


**Figure S20**. Volumetric water content (VWC) measurements from two locations at the field site in Council, Alaska. Near surface VWC in the field shows lower VWC than the column experiments in the winter months due to freezing. Summer months range from 0.5-0.7 VWC, which is comparable to soil column experiments. Data from Vladimir Romanovsky, William Cable, Kirill Dolgikh. 2022. Soil Temperature and Moisture, Council Road Mile Marker 71, Seward Peninsula, Alaska, beginning 2016. Next Generation Ecosystem Experiments Arctic Data Collection, Oak Ridge National Laboratory, U.S. Department of Energy, Oak Ridge, Tennessee, USA. Dataset accessed in 2023 at https://doi.org/10.5440/1581170.


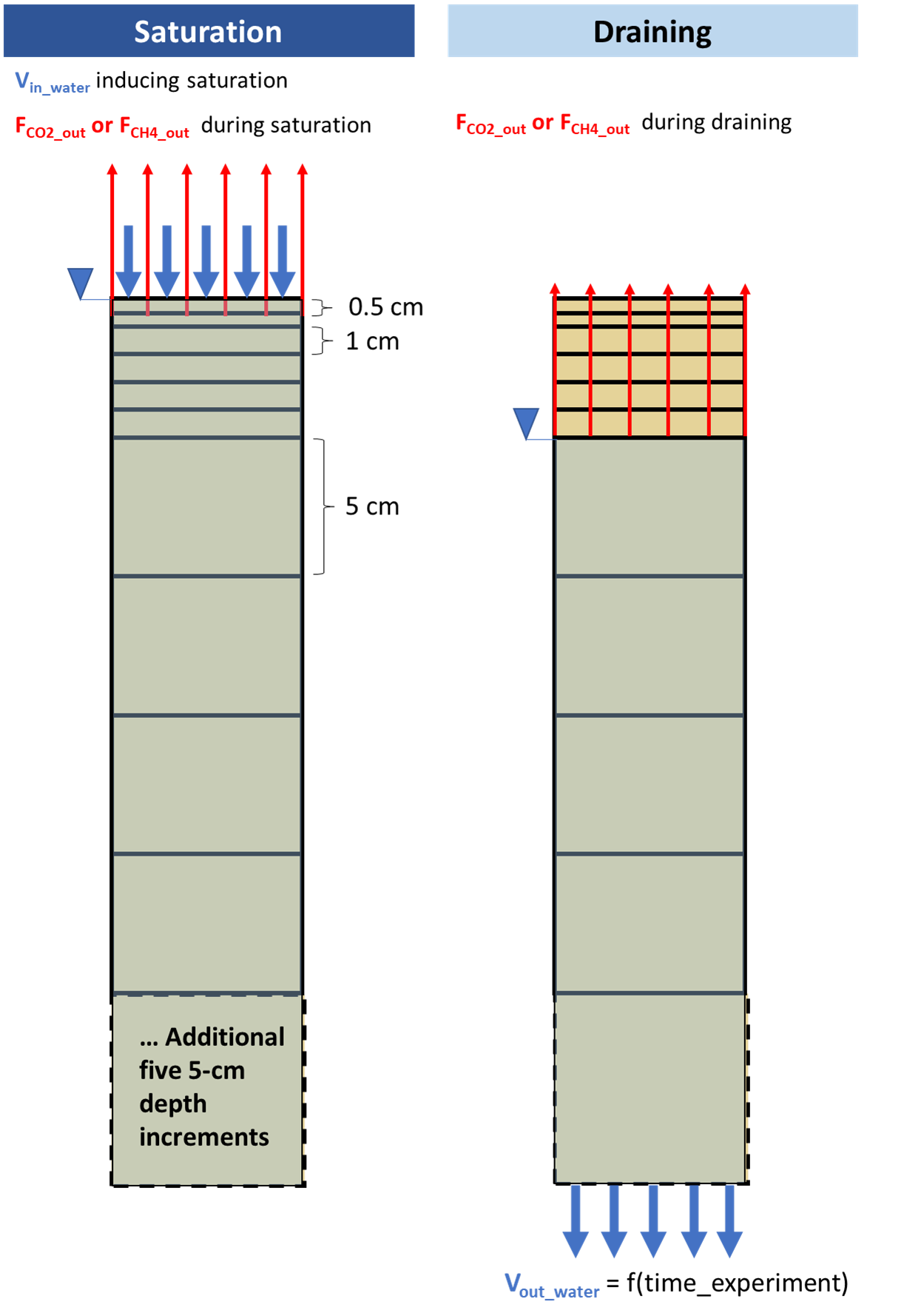


**Figure S21.** Model discretization and boundary conditions for simulations during saturation (left) and draining (right). V designates volumes of water and F designates fluxes of gases out of the top surface of the column. The decreasing water table during drained created unsaturated zones (tan color) with gas-phase diffusion.

# S3. Supporting References

1 Herndon, E. *et al.* Influence of iron redox cycling on organo-mineral associations in Arctic tundra soil. *Geochimica et Cosmochimica Acta* **207**, 210-231 (2017).

2 Patzner, M. S. *et al.* Iron mineral dissolution releases iron and associated organic carbon during permafrost thaw. *Nature communications* **11**, 1-11 (2020).

3 Amacher, M., Henderson, R., Breithaupt, M., Seale, C. & LaBauve, J. Unbuffered and buffered salt methods for exchangeable cations and effective cation‐exchange capacity. *Soil Science Society of America Journal* **54**, 1036-1042 (1990).

4 Loveland, P. & Digby, P. The extraction of Fe and Al by 0.1 M pyrophosphate solutions: a comparison of some techniques. *Journal of Soil Science* **35**, 243-250 (1984).

5 Coward, E. K., Thompson, A. T. & Plante, A. F. Iron-mediated mineralogical control of organic matter accumulation in tropical soils. *Geoderma* **306**, 206-216 (2017).

6 Chao, T. & Zhou, L. Extraction techniques for selective dissolution of amorphous iron oxides from soils and sediments. *Soil Science Society of America Journal* **47**, 225-232 (1983).

7 Lalonde, K., Mucci, A., Ouellet, A. & Gélinas, Y. Preservation of organic matter in sediments promoted by iron. *Nature* **483**, 198-200 (2012).

8 Filippova, N. & Bulyonkova, T. Notes on the ecology of Ascocoryne turficola (Ascomycota: Helotiales) in West Siberia. *Environmental dynamics and global climate change* **4**, 1-6 (2013).

9 Põlme, S. *et al.* FungalTraits: a user-friendly traits database of fungi and fungus-like stramenopiles. *Fungal diversity* **105**, 1-16 (2020).

10 Douglas, G. M. *et al.* PICRUSt2 for prediction of metagenome functions. *Nature biotechnology* **38**, 685-688 (2020).

11 Garber, A. I. *et al.* FeGenie: a comprehensive tool for the identification of iron genes and iron gene neighborhoods in genome and metagenome assemblies. *Frontiers in Microbiology* **11**, 37 (2020).

12 Sulman, B. N. *et al.* Simulated hydrological dynamics and coupled iron redox cycling impact methane production in an Arctic soil. *Journal of Geophysical Research: Biogeosciences* **127**, e2021JG006662 (2022).

13 Liu, H. & Lennartz, B. Hydraulic properties of peat soils along a bulk density gradient—A meta study. *Hydrological Processes* **33**, 101-114 (2019).

14 Gupta, S., Hengl, T., Lehmann, P., Bonetti, S. & Or, D. SoilKsatDB: global soil saturated hydraulic conductivity measurements for geoscience applications. *Earth System Science Data Discussions* **2020**, 1-26 (2020).

15 Roy Chowdhury, T. *et al.* Temporal, spatial, and temperature controls on organic carbon mineralization and methanogenesis in Arctic high-centered polygon soils. *Frontiers in Microbiology* **11**, 3414 (2020).
